# Self-pigmenting textiles grown from cellulose-producing bacteria with engineered tyrosinase expression

**DOI:** 10.1038/s41587-024-02194-3

**Published:** 2024-04-02

**Authors:** Kenneth T. Walker, Ivy S. Li, Jennifer Keane, Vivianne J. Goosens, Wenzhe Song, Koon-Yang Lee, Tom Ellis

**Affiliations:** 1 Imperial College Centre for Synthetic Biology, Imperial College London, London, UK; 2 Department of Bioengineering, Imperial College London, London, UK; 3 Modern Synthesis, London, UK; 4 Department of Aeronautics, Imperial College London, London, UK

**Keywords:** Biomaterials, Synthetic biology, Industrial microbiology

## Abstract

Environmental concerns are driving interest in postpetroleum synthetic textiles produced from microbial and fungal sources. Bacterial cellulose (BC) is a promising sustainable leather alternative, on account of its material properties, low infrastructure needs and biodegradability. However, for alternative textiles like BC to be fully sustainable, alternative ways to dye textiles need to be developed alongside alternative production methods. To address this, we genetically engineer *Komagataeibacter rhaeticus* to create a bacterial strain that grows self-pigmenting BC. Melanin biosynthesis in the bacteria from recombinant tyrosinase expression achieves dark black coloration robust to material use. Melanated BC production can be scaled up for the construction of prototype fashion products, and we illustrate the potential of combining engineered self-pigmentation with tools from synthetic biology, through the optogenetic patterning of gene expression in cellulose-producing bacteria. With this study, we demonstrate that combining genetic engineering with current and future methods of textile biofabrication has the potential to create a new class of textiles.

## Abstract (web summary)

Genetic engineering of material-producing bacteria creates pigmented products.

## Main

The textile and leather industry impacts the environment—contributing to greenhouse gas emissions from agricultural production and industrial processing, water pollution through tanning and dyeing and microplastic pollution from synthetic fiber shedding^1–3^. To reduce the impact of this industry, new sustainable biomaterials are under commercial development. These include mycelium and plant fiber-based leather alternatives^4–6^. These endeavors are the successful result of combining biological production with engineering and chemical processing to refine these natural biomaterials into alternative textiles. However, the industry has yet to employ genetic engineering of these material-producing organisms to take advantage of the sustainable methods biological systems use to enhance the physical and esthetic properties of biomaterials.

The field of engineered living materials (ELMs) uses the tools of synthetic biology to reprogram living cells at the DNA level to build new or enhanced biomaterials for specific applications^7,8^. Bacterial cellulose (BC) is a promising natural biomaterial, produced most effectively by bacteria in the Gram-negative genus *Komagataeibacter*^9^. In carbon-rich media, these bacteria polymerize and secrete linear chains of glucose. These chains then self-assemble into a dense interconnected mesh of cellulose fibers. This cellulose mesh, called a pellicle, floats at the air–water interface and envelops and protects the growing cells, like a biofilm^10^. Key to the industrial interest in BC, it can be grown quickly, cheaply and sustainably—a BC pellicle can be grown in 7–14 days, in high yields (>10 g l^−1^) and from waste feedstocks, such as rotten fruit juice, glycerol and molasses^11–13^. Additionally, BC has advanced material properties such as high tensile strength, high water-holding capacity and high purity^14,15^. These features have led to interest in using BC in high-end acoustic devices, as a battery separator membrane and in wound healing^16–21^. The ease of growing BC has also led to BC becoming an attractive prototype biomaterial for some in design and fashion who seek to speculate on methods of sustainable textile production^22^. The production of BC by culturable, low-risk bacteria also makes BC accessible to those seeking to modify it genetically using synthetic biology. Therefore, BC represents an ideal ‘blank slate’ for ELM research.

BC ELM research has focused on the genetic engineering of *Komagataeibacter* and other organisms such as *Saccharomyces cerevisiae* that can be cocultured with *Komagataeibacter*. The use of incorporated *S. cerevisiae* has allowed for the production of pellicles that can sense and respond to chemical and light stimuli^23^. To facilitate the genetic engineering of *Komagataeibacter*, a modular gene cloning tool kit, the Komagataeibacter tool kit (KTK), has been created and characterized using *Komagataeibacter rhaeticus*. Contributions to this synthetic biology tool kit include a selection of modular DNA parts, such as constitutive and inducible promoters, vectors and fluorescent markers^24–26^. Such parts have been used to make engineered *Komagataeibacter* that can produce alternative polymers, such as chitin, hyaluronic acid and curli fibers^26–28^. Additionally, engineered multicellular communication has been established in a pellicle, through cell-to-cell signaling between *K. rhaeticus* cells using quorum sensing molecules^29^. However, despite these achievements, genetic engineering has yet to be used to further the development of BC as a sustainable biomaterial in textiles and fashion.

Biomaterials in nature, such as hair and skin, use incorporated cells to produce pigments that color the biomaterial in situ in a low-impact sustainable manner. The parallel process used in industrial coloring of fabric materials, textile dyeing, requires chemical reactions and is highly damaging to the environment. Inspired by natural pigment production, we set out to engineer a self-pigmenting BC material through the genetic engineering of *K. rhaeticus*. Black dye is one of the most consumed dyes in the world, and one of the most difficult to recreate using sustainable dyeing approaches^30,31^. We decided to engineer the biosynthesis of the dark melanin pigment, eumelanin, into *K. rhaeticus*. Eumelanin, a ubiquitous pigment found across biological kingdoms, is stable in high heat and over long time spans^32^. Crucially, eumelanin has low water solubility, a property shared by many common dyes, such as indigo, that contributes to the color fastness of a pigment^33^. Additionally, eumelanin also offers several other interesting properties, such as electrical conductivity, broadband light and UV absorption and protection from ionizing radiation^34–38^.

We demonstrate here that the production of pigmented cellulose from *K. rhaeticus* can be produced in large enough quantities for the prototyping of fashion products. Furthermore, we illustrate the potential of combining melanin biosynthesis with other synthetic biology tools, through the optogenetic patterning of gene expression in growing BC pellicles.

## Results

### Melanin production from *K. rhaeticus*

**Fig. 1:**
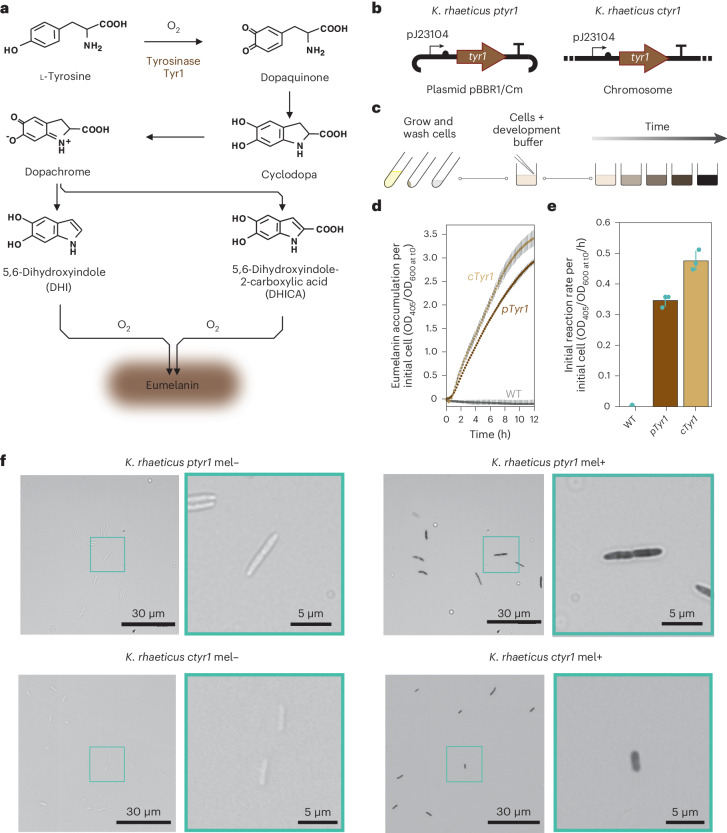
Eumelanin production from *K. rhaeticus* tyrosinase expression. **a**, Chemical pathway of eumelanin production from l-tyrosine. The first step involves the hydroxylation of l-tyrosine to l-DOPA that is catalyzed by tyrosinase—here acting as a monophenol mono-oxygenase. This step is then followed by the catalysis of l-DOPA to dopaquinone, which is catalyzed by the diphenolase activity of Tyr1. The remaining steps in the pathway occur spontaneously in the presence of oxygen, leading to the generation of eumelanin. **b**, Genetic construct maps for the following two *K. rhaeticus* tyrosinase expression strains: *K. rhaeticus ptyr1* and *K. rhaeticus ctyr1*. Both constructs use the same constitutive promoter (pJ23104), RBS (B0034) and terminator (L3S1P00). *K. rhaeticus ptyr1* uses a plasmid with a pBBR1 origin of replication and a chloramphenicol resistance cassette. **c**, A two-step process for eumelanin production from *K. rhaeticus* grown in shaking conditions. Strains are grown in HS-glucose media, washed and resuspended with PBS to remove spent media before being mixed with melanin development buffer. **d**, Tyr1-producing strains are assayed for eumelanin production. Eumelanin production per initial cell was determined by measuring OD_405_ over 12 h, divided by the initial OD_600_ of each well at time point 0. Points show the mean of three biological replicates. Error bars are the s.d. of three biological replicates. **e**, Initial reaction rate per initial cell was determined by measuring the gradient of eumelanin accumulation per initial cell from 50 to 170 min after the start of measurement. Bars show the means of three biological replicates of each strain, while error bars show the s.d. **f**, Optical microscopy images of *K. rhaeticus ptyr1 and K. rhaeticus ctyr1* before (mel−) and after melanin development (mel+). A zoomed-in example of a single cell is shown with a cyan outline. Images shown are representative of four random images taken for each strain and treatment. Source data

Recombinant production of eumelanin has been demonstrated in *Escherichia* *coli* and *Vibrio natrigens* in the pursuit of diverse applications like bioremediation and bioelectronics^39–42^. The bacterial production of eumelanin requires only a single enzyme (tyrosinase) that catalyzes the oxidation of l-tyrosine to dopaquinone—the rate-limiting step in eumelanin synthesis^32^. In oxygenated and temperate conditions, dopaquinone spontaneously converts into eumelanin via several steps (Fig. 1a). The prokaryotic tyrosinases that have been tested in a recombinant context are, MelA from *Rhizobium etli* and Tyr1 from *Bacillus megaterium*^41,43^. We decided to focus on Tyr1 for this study, due to its smaller size and proven use in nonmodel organisms. Using our KTK system for modular cloning, we created the following two constitutive *K. rhaeticus* Tyr1 expression strains: the plasmid-based *K. rhaeticus ptyr1* and the chromosomally integrated *K. rhaeticus ctyr1* (Fig. 1b). Both strains used identical upstream and downstream DNA parts around the *tyr1* coding sequence. The promoter used upstream of *tyr1* for both constructs was the synthetic constitutive promoter pJ23104, which was previously found to have the strongest expression strength of a library of promoters characterized in *K. rhaeticus*^26^.

**Extended Data Fig. 1:**
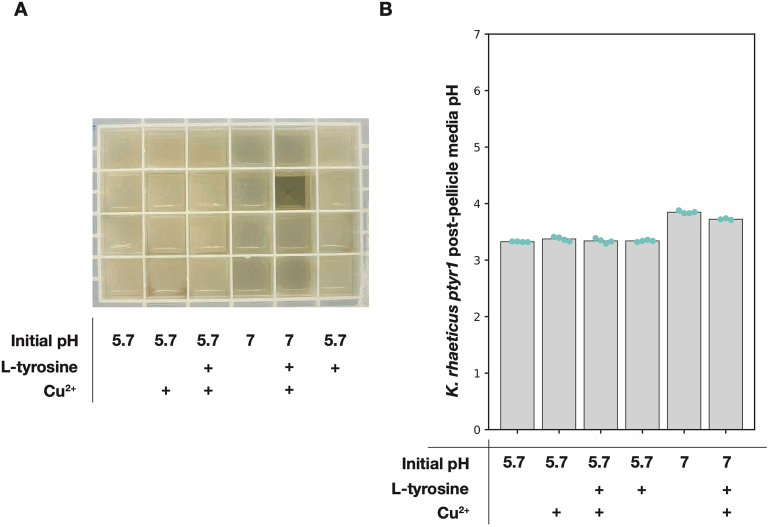
Post-pellicle growth media pH of *K. rhaeticus ptyr1*. **a**, Pellicles produced after 7 days growth at 30 °C in a 24-well deep well plate. Each plate column contains 4 replicated media compositions. Buffered HS-glucose media pH values before growth are noted beneath, as well as additional components required for melanin production. **b**, pH values of media underneath the pellicle after 7 days growth at 30 °C. Data shown are average and standard deviation of 4 replicates, except for media set to pH 7 with L-tyrosine and Cu, where only 3 replicates produced pellicles.

Melanin synthesis by Tyr1 is sensitive to pH—only occurring readily at pH values above 7 (ref. ^43^). This conflicts with the growth of *K. rhaeticus*, which, as an acetic acid bacteria, acidifies its culture media during growth by the production of organic acids such as gluconic and acetic acid^44,45^. Indeed, we found that *K. rhaeticus ptyr1* pellicles grown in Hestrin–Schramm glucose (HS-glucose) media buffered to pH 5.7 and with the necessary substrate and cofactors for eumelanin production (0.5 g l^−1^ l-tyrosine and 10 μM CuSO_4_), displayed no pigmentation during growth (Extended Data Fig. 1a)^46^. We measured the acidification of the growth media after pellicle production, which demonstrated that the culture pH had lowered to below pH 4, even when the initial media pH was buffered higher to pH 7 (Extended Data Fig. 1b). These results suggested that we would need to separate pellicle growth from eumelanin production.

**Extended Data Fig. 2:**
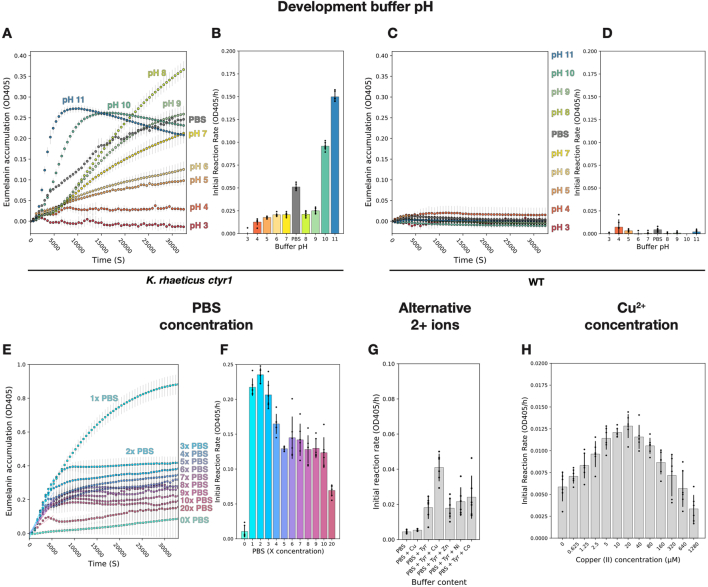
Characterization of melanin production rates in differing melanin development buffer formulations. **a**, Time course experiment of eumelanin accumulation at OD_405_ from *K. rhaeticus tyr1* in a range of pH values. Phosphate buffered saline (PBS) set to 7.4 containing 0.5 g/L L-tyrosine and 10 μM CuSO_4_ is shown in gray. Points represent the mean while error bars represent the SD of 6 replicates. **b**, Average initial reaction rates of melanin accumulation from *K. rhaeticus tyr1* in buffers of differing pH values. Reaction rates are derived from the gradient of OD_405_ over the first 140 minutes of the experiment. Colors match those in panel a. Bars represent the mean rate while error bars represent the SD of 6 replicates. **c**,**d**, Time course experiment of melanin accumulation and initial reaction rates of melanin accumulation from *K. rhaeticus* WT. Points and bars represent the mean while error bars represent SD of 6 technical replicates. **e**, Time course experiment of melanin accumulation at OD_405_ from *K. rhaeticus tyr1* in differing PBS concentrations. A PBS concentration of 0x corresponds to ddH_2_O containing only melanin development substrates (L-tyrosine and CuSO_4_) while a PBS concentration of 1× corresponds to 137 mM NaCl, 2.7 mM KCL, 10 mM Na_2_HPO_4_ and 1.8 mM KH_2_PO_4_. Melanin development substrates are maintained at 0.5 g/L tyrosine and 10 μM CuSO_4_ for all PBS concentrations. Points represent the mean while error bars represent SD of 6 replicates. **f**, Average initial reaction rates of melanin accumulation from *K. rhaeticus tyr1* in differing PBS concentrations. Reaction rates are derived from the gradient of OD_405_ over the first 140 minutes of the experiment. Error bars represent the SD of 6 replicates. **g**, Average initial reaction rates of melanin accumulation from *K. rhaeticus tyr1* in buffers containing different metal (II) ions. Ions present in solution at 20 μM. Error bars represent the SD of 6 replicates. **h**, Average initial reaction rates of melanin accumulation from *K. rhaeticus tyr1* in buffers containing different copper (II) concentration. Error bars represent the SD of 6 replicates.

We, therefore, decided to employ a two-step approach to produce melanin from *K. rhaeticus*. The first step would involve growing Tyr1-expressing *K. rhaeticus* under normal growth conditions and the second step would involve removing the spent culture media and replacing it with a buffered solution with the reagents required for melanin synthesis (Fig. 1c). For the buffered solution, which we refer to as melanin development buffer, we chose to use PBS, buffered to pH 7.4, containing 0.5 g l^−1^ l-tyrosine and 10 μM CuSO_4_. We then tested this approach with both of our Tyr1-expressing strains. To enable the quantification of eumelanin production, we assayed *K. rhaeticus* cells that had grown in shaking conditions with cellulase added to the media, which prevents pellicle formation. We measured eumelanin production in the melanin development buffer at OD_405_ over 12 h (Fig. 1d). Both Tyr1-expressing *K. rhaeticus* strains were able to produce eumelanin in the development buffer. The melanin production rate per initial cell was higher for the integrated tyrosinase strain *K. rhaeticus ctyr1* (0.48 ± 0.03 OD_405_/OD_600_/h) versus the plasmid-based *K. rhaeticus ptyr1* (0.35 ± 0.02 OD_405_/OD_600_/h; Fig. 1e). We also used this same experimental approach to assay the effect on melanin production of changing the pH, salt concentration, oxidation state (II) metal ion and copper (II) concentration of the melanin development buffer (Extended Data Fig. 2a–h). Interestingly, we found alkaline buffer conditions (>pH 8) led to a more rapid production of eumelanin than neutral conditions. However, the production rate in alkaline buffer conditions also slowed rapidly, leading to overall lower melanin accumulation than at pH 8. This result conflicted with previous in vitro studies on Tyr1 that suggested an optimal pH value of 7 (ref. ^43^). The same study did state, however, that l-DOPA may spontaneously convert into dopachrome at pH values above 7.5. The in vivo result seen here may reflect a spontaneous conversion of built-up l-DOPA inside the cells, which may suggest Tyr1 has some level of monophenolase activity (l-tyrosine to l-DOPA) during the *K. rhaeticus* growth stage, despite a lack of visible melanin production.

**Extended Data Fig. 3:**
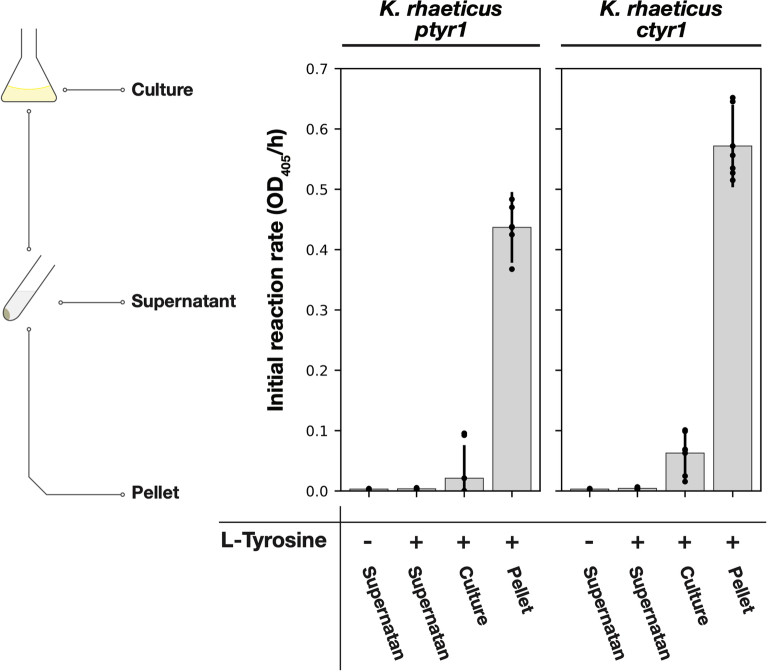
Assaying cell supernatant for tyrosinase activity. A 12-hour time course experiment of eumelanin accumulation at OD_405_ from *K. rhaeticus tyr1* and *K. rhaeticus* culture, centrifuged cell pellets and remaining supernatant. Presence or absence of L-tyrosine at 0.5 g/L in each assay sample is shown below each bar. Average initial reaction rates shown as bars were derived from the gradient of OD_405_ from 50–190 minutes after the start of the assay. Error bars represent the standard deviation of 6 replicates.

We then looked at how *K. rhaeticus* cells had changed after exposure to the melanin development buffer. Using light microscopy, we found that *K. rhaeticus ptyr1 and ctyr1* cells exposed to the development buffer appeared visibly darker, suggesting eumelanin production may be occurring intracellularly (Fig. 1f). We also subjected supernatants from *K. rhaeticus ptyr1 and ctyr1* cultures to the melanin production assay and found no significant difference in melanin accumulation rate between supernatant samples with and without l-tyrosine, indicating that extracellular Tyr1 presence in shaking cultures was minimal (Extended Data Fig. 3). This was as expected, given that the Tyr1 protein did not contain secretion or translocation tags. However, as the onset of eumelanin production requires the cells to be immersed in a neutral pH buffer, this also suggests that the cytoplasmic pH of *K. rhaeticus* may also become acidic during growth. Indeed, other acetic acid bacteria show adaptations suggestive of acidic internal conditions^44,47^.

### Pigmenting BC through *K. rhaeticus* eumelanin production

**Fig. 2:**
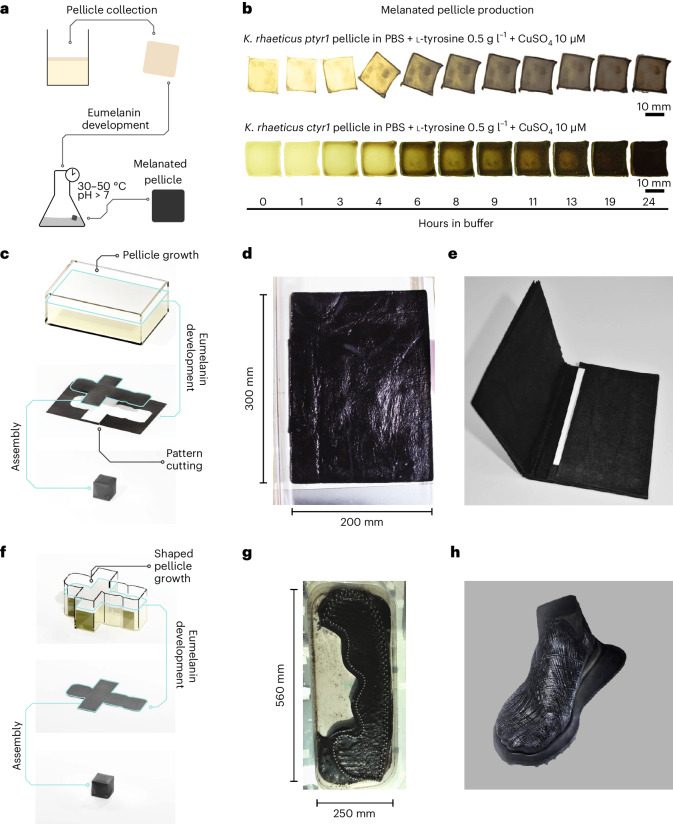
Using *tyr1* expressing *K. rhaeticus* to produce melanated BC. **a**, The production process for melanated BC involves two steps. Tyrosinase-expressing *K. rhaeticus* are grown in static conditions to produce a pellicle. Once grown, this is collected and placed in development buffer and incubated with agitation between 30 °C and 50 °C until the material reaches the desired shade. **b**, Images show a time lapse of the progression of eumelanin accumulation over 24 h at 30 °C for *K. rhaeticus ptyr1* and *K. rhaeticus ctyr1* pellicles placed in development buffer. **c**, Pellicle production can be conducted in standardized containers to produce sheets of BC from which pattern pieces can be cut out and assembled. **d**, A *K. rhaeticus* c*tyr1* pellicle, grown in a 300 × 200 mm container, after eumelanin development step. **e**, A finalized wallet prototype, cut and assembled from two pressed and dried melanated BC sheets. **f**, Pellicle production can also occur in shaped containers, producing BC preshaped to the 2D pattern of the final pattern piece. **g**, A *K. rhaeticus ptyr1* pellicle grown in a shaped container, after eumelanin development. Metal pins seen within the perimeter of the pellicle hold a network of woven thread that becomes integrated into the pellicle during growth. **h**, A finalized shoe upper prototype produced from a melanated shaped BC sheet with integrated yarn that has been wrapped around a foot-shaped last and placed on a shoe sole.

**Extended Data Fig. 4:**
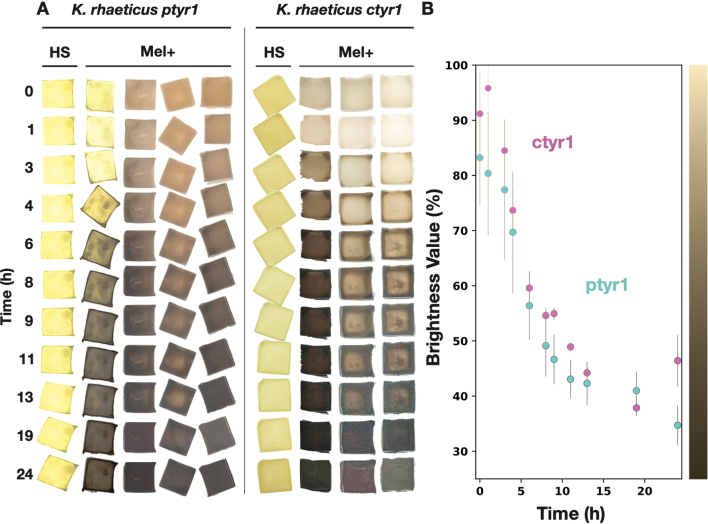
Timelapse of pellicle melanin production. **a**, Timelapse images of *K. rhaeticus ptyr1* and *ctyr1* pellicles. Pellicles labeled under HS, were incubated in HS media, while those pellicles labeled under Mel+ were incubated in PBS with 0.5 g/L L-tyrosine and 10 μM CuSO_4_. **b**, Graph displays the average brightness value of the pellicle images over 24 hours as melanin production occurs. Brightness value is derived from converting images from the RGB (red, green, blue) color space into the HSB (hue, saturation, brightness) color space, where the brightness value represents the value of a color as a percentage between black and peak luminosity, 0–100% respectively. Points represent the means while error bars represent the standard deviation of brightness values from 3 separate pellicles from *K. rhaeticus* *ctyr1* and 4 separate pellicles from *K. rhaeticus* *ptyr1*.

**Extended Data Fig. 5:**
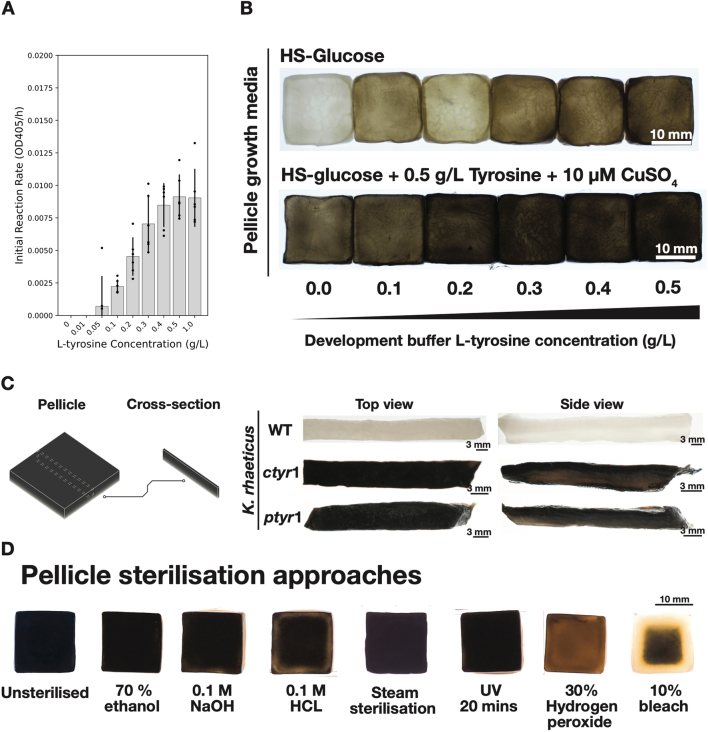
Melanin pigmentation strength and stability during sterilization. **a**, Average initial reaction rates of melanin accumulation from *K. rhaeticus tyr1* in PBS buffer with 10 μM CuSO_4_ with differing concentrations of L-tyrosine. An L-tyrosine concentration of 1 g/L is above the maximum solubility of tyrosine in water at 25 °C—OD_405_ readings at this concentration and above become inaccurate due to suspended undissolved L-tyrosine. Error bars represent the standard deviation of 6 replicates and mean reaction rates shown as bars are derived from the first 140 minutes of the reaction. **b**, *K. rhaeticus ptyr1* pellicle pigmentation after 24 hours in a shaking melanin development bath at 30 °C containing differing L-tyrosine concentration. The top row shows pellicles from *K. rhaeticus ptyr1* grown in HS-glucose media without L-tyrosine and CuSO_4_ while the bottom row shows pellicles grown in HS-glucose media with L-tyrosine and CuSO_4_. **c**, Images of melanated and unmelanated pellicle cross-sections from *K. rhaeticus* WT, *ptyr1* and *ctyr1*. Top and side views are shown with backlighting to clarify differences in melanin pigmentation. **d**, Effects of various sterilization methods on melanin stability in melanated pellicles from *K. rhaeticus ptyr1*. Pellicles had been bathed in development buffer containing 0.5 g/L L-tyrosine and 10 μM CuSO_4_ for 24 hours. Pellicles were exposed to sterilizing conditions for 2 hours, except gel doc UV light, to which the pellicle was exposed to for 20 minutes.

**Extended Data Fig. 6:**
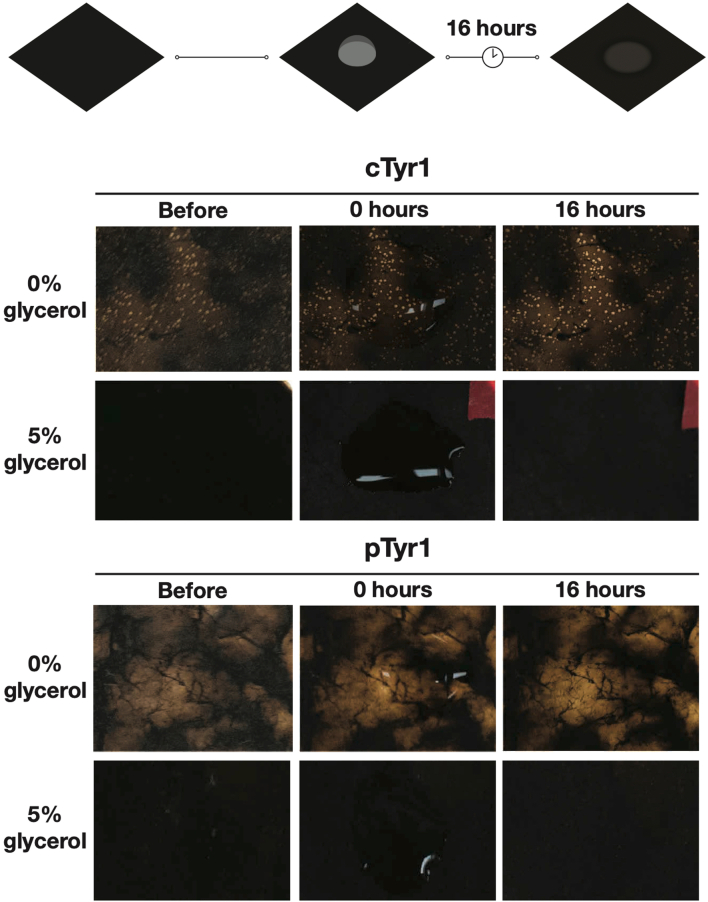
Testing melanin BC color fastness with water spotting test. The top illustration demonstrates the water spotting process. Photographs of dried melanated BC from both *K. rhaeticus* strains, with and without glycerol treatment are shown at three time points: before water spotting, immediately after water spotting and 16 hours after water spotting. Images are representative of 3 water spotting tests for each pellicle and treatment.

Having shown eumelanin production from *K. rhaeticus* cells expressing *tyr1*, we next wanted to demonstrate that eumelanin production could effectively pigment BC. To do so, we applied the same two-step process to *K. rhaeticus ptyr1* and *ctyr1* static cultures that had grown pellicles (Fig. 2a). Following 24 h of shaking incubation at 30 °C in the development buffer, the pellicles changed appearance from a pale yellow to a brownish black, demonstrating eumelanin pigmentation of BC (Fig. 2b). After quantifying the visual darkening of *K. rhaeticus ptyr1 and ctyr1* over time, in the conditions tested, we found they reached peak visible darkness after 19 h (Extended Data Fig. 4). Additionally, by reducing l-tyrosine concentration in the development buffer, we could slow the rate of melanin production (Extended Data Fig. 5a), allowing us to vary how pigmented a BC pellicle becomes and thus generate material in a range of brown shades (Extended Data Fig. 5b). From thin cross-section slices of hydrated melanated pellicles, we observed that melanin pigmentation was darkest at the top of the pellicle (Extended Data Fig. 5c). We also found that including 0.5 g l^−1^ l-tyrosine in both culture medium and the melanin development buffer led to the darkest pellicles, presumably as this allows l-tyrosine levels and eumelanin precursors to build-up in the cells during growth. Crucial to the use of melanated BC outside of laboratory contexts is that the pigment persists through sterilization. We found that both high-pressure steam and ethanol sterilization worked well to preserve pigmentation (Extended Data Fig. 5d). As expected, sterilization by oxidizing compounds, such as sodium hypochlorite bleach, led to a rapid loss of melanin pigmentation. Finally, we tested the color fastness of melanated BC to water spotting—a common test for leather stains. We found the color of melanated BC to be highly stable with no visible discoloration due to water spotting (Extended Data Fig. 6).

Due to high yields of material being produced from simple static growth cultures, microbial production of BC is very amenable to scale-up, enabling the amount of BC required to build real products to be achieved with minimal infrastructure investment. This has made BC attractive to producers at both industrial and cottage-industry scales, especially as a vegan alternative to leather for use in clothing and accessories. With this in mind, we wanted to demonstrate that we could scale the growth of Tyr1-expressing *K. rhaeticus* to produce functionally useful quantities of pigmented BC. For this, we considered two approaches to BC production. In our first approach, we sought to produce a standardized sheet of melanated BC, from which a nonwoven textile pattern (that is template) could be cut and assembled (Fig. 2c). To aid in the large-scale growth of BC, we switched to growth media containing coconut water, 1% ethanol and 1% acetic acid. This media is used in industrial environments to grow *K. rhaeticus* and maximize BC production^48^. We grew *K. rhaeticus ctyr1* in a 300 × 200 mm tray and after 10 days of growth, we collected the pellicle and let it undergo eumelanin production until it had taken on a deep black color (Fig. 2d). The melanated BC sheet was then sterilized by autoclave, pressed flat and dried. The BC sheet retained its color throughout this process (Supplementary Video 1). A wallet pattern was then cut from two of these melanated sheets, and the pattern pieces were sewn together with thread to make a functioning melanated BC wallet (Fig. 2e).

In our second approach, we took advantage of how pellicle growth follows the air–water interface and grows in the same shape as the culturing vessel (Fig. 2f). Using *K. rhaeticus ptyr1*, we grew a pellicle in a bespoke culture vessel, in the shape of a shoe upper pattern piece. This culture vessel contained a loom-like apparatus holding a network of strung Lyocell (TENCEL) threads, located at the air–water interface to allow these threads to be incorporated into the growing *K. rhaeticus ptyr1* pellicle. After 14 days of growth, the final pellicle and apparatus were removed from the culture media and placed into development buffer. After 48 h of gentle shaking at 30 °C, the pellicle had taken on a deep black color (Fig. 2g). The pellicle was then sterilized by ethanol bath and soaked in a 5% glycerol solution, before being removed from the apparatus and wrapped in around an epoxy shoe last (that is a foot-shaped mold) and allowed to dry (Fig. 2h).

It is still the case that melanated BC, along with standard non-GMO BC materials, require additional processing and additives to meet the expectations of current material properties for alternative leathers. Nonetheless, we believe that both the shoe upper and wallet demonstrate that our engineered strains can grow and self-pigment at scales large enough to produce prototype fashion pieces, which can demonstrate the possible form and esthetics of melanated BC. These pieces also demonstrate the positive outcomes of collaboration between scientists and designers in the pursuit of creating new ELMs. As users of new biomaterial-based textiles, designers have a key role in demonstrating and publicizing the features of a new material and can give constructive feedback to scientists on any limitations and how the materials could be improved, particularly in order for their end-of-life to become more sustainable.

### Characterization of melanated cellulose

**Fig. 3:**
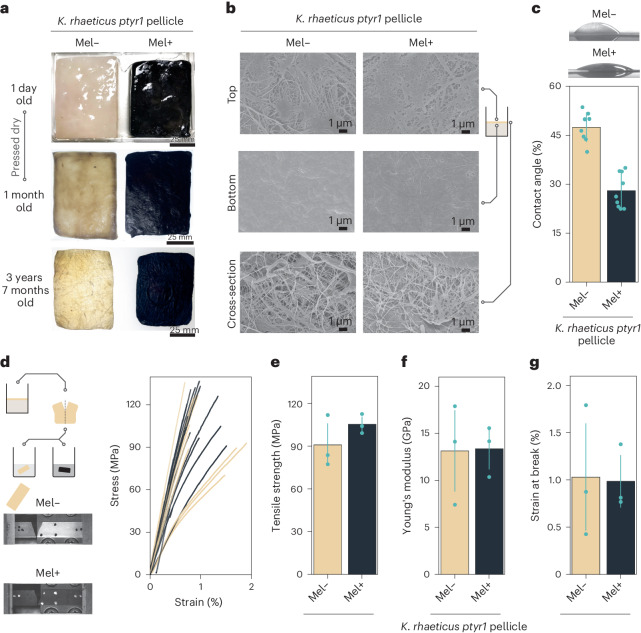
Properties of melanated BC. **a**, A melanated (mel+) and unmelanated (mel−) swatch, from the same original *K. rhaeticus ptyr1* pellicle. These swatches had been dried and used as demonstration pieces. **b**, SEM of mel+ and mel− *K. rhaeticus ptyr1* BC. The top and bottom surfaces pertain to the air-facing and media-facing pellicle surfaces, respectively. Images shown are representative of at least five images per condition. **c**, The sessile drop method was used to measure the contact angle on *K. rhaeticus ptyr1* mel− (beige) and mel+ (black) pellicles. An unpaired *t* test result gave a value of *P* < 0.005 and error bars represent s.d. from eight mel− and nine mel+ drop measurements. Representative water drop shapes for mel+ and mel− pellicles are shown above the graph. **d**, Comparative tensile tests of melanated and unmelanated BC sheets were conducted using BC sheets prepared from halves of the same *K. rhaeticus ptyr1* pellicle. Representative images of BC breaks are given for mel− and mel+ BC as well as stress–strain curves of the technical repeats from three biological replicates of mel+ and mel− pellicles. **e**–**g**, Tensile strength (**e**), Young’s modulus (**f**) and strain at break (**g**) for mel+ and mel− pellicles—the *P* values from paired *t* tests between mel+ and mel− BC were 0.17, 0.92 and 0.85, respectively. Error bars show s.d. from three biological replicates and each biological replicate is the average of three or more technical replicates. Source data

A swatch of melanated cellulase produced by *K. rhaeticus ptyr1* was actively used as a demo piece for 42 months and maintained its pigmentation throughout (Fig. 3a), demonstrating that the color was resilient over time. As well as color, we were curious to know how eumelanin production may have impacted the other material properties of BC. To investigate this, we first checked to see whether eumelanin had altered the BC surface using scanning electron microscopy (SEM). We compared the top and bottom surfaces, as well as the cross-sections, of melanated and unmelanated *K. rhaeticus ptyr1* pellicles (Fig. 3b). The SEM images indicated minimal structural differences between melanated and unmelanated pellicles. The uneven surface morphology on the top surface images is attributed to leftover embedded cells, in contrast to smoother morphology on the bottom surface. In addition, cross-sections of the melanated and unmelanated ptyr1 pellicles show minimal differences in the porosity of the BC nanofibril network. To further study the surface material properties of melanated cellulose, we conducted wettability testing using the static sessile drop method (Fig. 3c). Using pellicles grown from *K. rhaeticus ptyr1*, we observed that the melanated pellicle had increased surface wettability, with an average contact angle of 28° compared to 47° for the unmelanated pellicle.

One of the most attractive features of BC for the industry is its high tensile strength; therefore, it is important to know whether melanation interferes with or enhances the strength of the BC nanofibril network. We carried out tensile testing using both melanated and unmelanated pellicles. For consistency, we prepared a paired set of BC samples, by splitting each grown pellicle in half and developing eumelanin in only one half of each pellicle. Both halves were heat pressed to consolidate the BC nanofibril network into dried BC sheets (Fig. 3d). The average tensile strength values were 91 MPa and 105 MPa for unmelanated and melanated pellicles, respectively (Fig. 3e). For Young’s modulus, the values were 13.7 GPa and 13.9 GPa for unmelanated and melanated pellicles, respectively (Fig. 3f). For the strain at break, the values were 1.02% and 0.98% for unmelanated and melanated samples, respectively (Fig. 3g). The material properties of all samples tested fell within the expected ranges of 70–300 MPa for BC tensile strength and 5–17 GPa for Young’s modulus^31^. A paired *t* test showed that the dried BC from melanated and unmelanated pellicles did not possess significant statistical differences in tensile material properties.

### Patterning eumelanin output

**Fig. 4:**
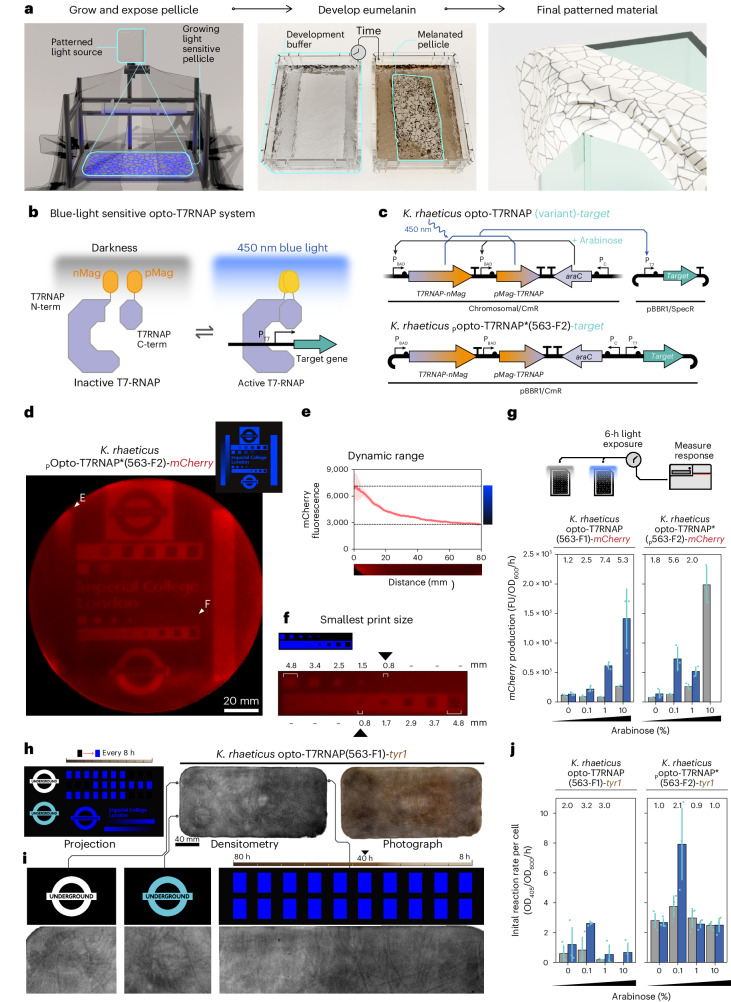
Functional optogenetics in *K. rhaeticus*. **a**, Proposed procedure to make patterned BC through optogenetics. **b**, The Opto-T7RNAP system uses a split T7-RNA polymerase, made blue light activatable via fusion with photo-sensitive magnet proteins. **c**, Genetic arrangements of *K. rhaeticus* optogenetic strains. Expression of the split T7RNAP genes is induced with arabinose. **d**, Red fluorescence scan of the top surface of a blue light exposed wet *K. rhaeticus* pOpto-T7RNAP*(563-F2)*-mCherry* pellicle (diameter = 150 mm). Graphic on top right shows the image projected onto the pellicle during growth. Pellicle shown is representative of two patterned pellicle repeats. **e**, The right of the projected image contained a gradated strip, from minimum to maximum light let through. Data show the intensity of red fluorescence seen in the pellicle against this gradated strip. The s.d. of pixel intensity for each horizontal slice is shown in pink. Black dotted line represents the intensity of unexposed pellicle regions. **f**, Smallest projected mark on the exposed pellicle. **g**, Characterization of optogenetics constructs with *mCherry* target gene under differing arabinose percentage (wt/vol) concentration. Bars (blue, exposed and gray, unexposed) show mean increase in red fluorescence after 6 h normalized by OD_600_. Error bars show s.d. of three biological replicates. Fold difference between exposed and unexposed cells is shown above, except in instances of poor growth. **h**, Comparison between projection video and the resulting wet *K. rhaeticus* Opto-T7RNAP(563-F1)-*tyr1* pellicle after eumelanin development (dimensions = 300 × 170 mm). Rectangles (black and blue) at top of projection video are timed to appear to aid in calculating minimum light exposure time. Densitometry scan and photograph of top surface of pellicle are shown. **i**, Zoomed-in sections of a densitometry scan of the *K. rhaeticus* Opto-T7RNAP(563-F1)-*tyr1* pellicle. Black triangle points to the sixth rectangle, indicating a 40-h required exposure time. **j**, Optogenetic construct characterization with *tyr1* under differing arabinose induction. The bars (blue, exposed and gray, unexposed) show the mean and s.d. of three biological replicates of initial (0–100 min) reaction rate of eumelanin production measured at OD_405_, normalized to the number of initial cells at OD_600_ at time point 0. Source data

Beyond coloring, textile processing can also involve patterning a textile. To showcase the capability of genetically engineered self-pigmented BC nonwoven textiles, we set out to establish spatial control of gene expression. We determined that the most flexible and precise way to pattern gene expression would be through engineered optogenetics and proposed a procedure for making patterned BC with light (Fig. 4a). To engineer a light-sensitive strain of *K. rhaeticus*, we chose to implement the blue-light-sensitive T7-RNA polymerase (Opto-T7RNAP) system originally designed for use in *E. coli* by Baumschlager et al.^49^ (Fig. 4b). We surmised that the Opto-T7RNAP system would be one of the simplest optogenetic systems to implement in a nonmodel organism like *K. rhaeticus*^50^. This view was based on the orthogonality of T7-RNA polymerase transcription, the lack of membrane-bound light-sensing components and the use of ubiquitous flavin adenosine dinucleotide (FAD) as a chromophore, which eliminates the need for heterologous chromophore biosynthesis genes.

**Extended Data Fig. 7:**
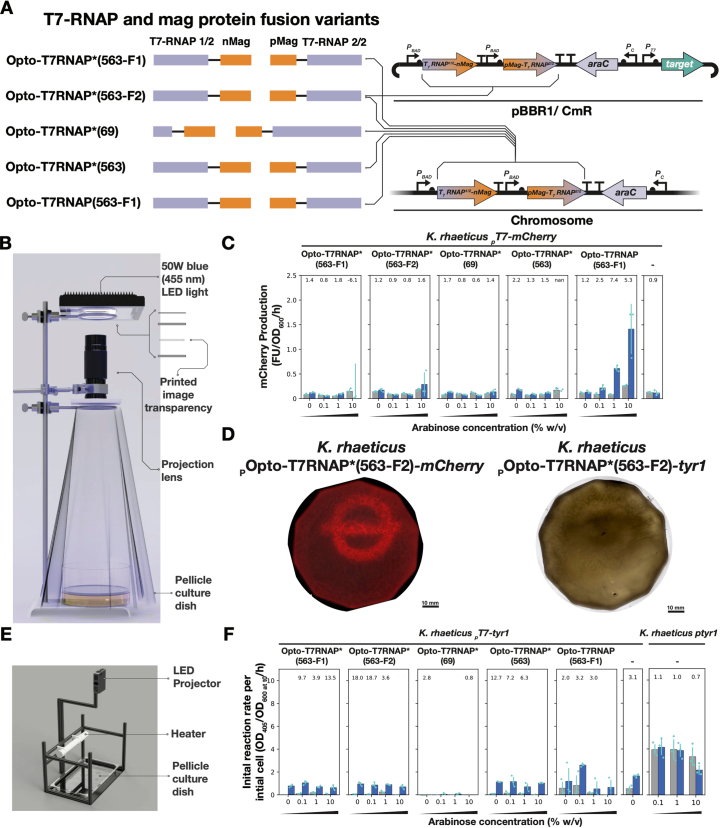
Additional optogenetic data. **a**, Five variants of the opto-T7RNAPs system were integrated into the *K. rhaeticus* chromosome. Only Opto-T7RNAP*(563-F2) was used to create the plasmid-based arrangement. **b**, A rig for projecting light onto a growing pellicle. Blue light from LED floodlight passes through an acetate transparency, on which is a printed image. The light that passes through this image is then focused and projected beneath onto the growing pellicle. Overall assembly is 620 mm tall. **c**, Blue-light response from all tested integrated constructs that were transformed with an *mCherry* target gene plasmid. Bars (blue = exposed, gray = unexposed) show average increase in red fluorescence after 6 hours normalized by OD_600_. Bars show mean rate while error bars show standard deviation of 3 biological replicates placed on the same plate. Fold difference between exposed and unexposed cells is shown at top. **d**, Comparison of *K. rhaeticus*_p_Opto-T7RNAP*(563-F2)-*mCherry* pellicle fluorescence scan and _p_Opto-T7RNAP*(563-F2)-*tyr1* pellicle photograph. Both pellicles are produced using identical methods. **e**, Commercial projector-based assembly for patterning blue light on to a growing pellicle. **f**, Characterization data from tested genome-integrated constructs that were transformed with a *tyr1* target gene plasmid. Fold difference is given above the bars, but not shown when rates are 0 or below. Bars show mean rate while error bars show standard deviation of 3 biological replicates placed on the same plate.

To implement the Opto-T7RNAP system in *K. rhaeticus*, we tested two arrangements of the necessary DNA parts. We selected the variant with the highest fold change between light and dark states mentioned in ref. ^49^ (Opto-T7RNAP*(563-F2)) as a basis of a plasmid-based version, where the light-sensitive T7RNAP genes and light-regulated target gene were placed on the same plasmid (Extended Data Fig. 7a). In an alternative arrangement, to test the variants of the Opto-T7RNAP system produced by Baumschlager et al.^49^, we integrated the light-sensitive T7RNAP variant genes into the *K. rhaeticus* chromosome. These *K. rhaeticus* T7RNAP variant strains were then transformed with a separate plasmid encoding the target gene (Fig. 4c). In both arrangements, the two Opto-T7RNAP light-sensitive genes were regulated by the P_BAD_ promoter, which had been previously shown to function in *K. rhaeticus* when induced with arabinose at a concentration of 2% (wt/vol)^25^. The arabinose regulator gene, *araC*, was placed downstream of the integrated two Opto-T7RNAP light-sensitive genes in both the chromosomal and plasmid arrangements.

We then tested whether we could pattern gene expression in *K. rhaeticus* using the Opto-T7RNAP system. To simplify this process, we started with our target gene being a fluorescent reporter gene that produces the red fluorescent protein (RFP) mCherry. We constructed a projection device that could project an image onto the surface of culture liquid as it grows a pellicle (Extended Data Fig. 7b). We inoculated *K. rhaeticus*_p_Opto-T7RNAP*(563-F2)-*mCherry* in the culturing vessel of this device, and once a thin pellicle had formed, we exposed this nascent pellicle to a projected image for 72 h. The collected pellicle showed successful pattering of *mCherry* expression (Fig. 4d). The pellicle showed a 2.54× fold change in fluorescence between the least and most exposed section of the pellicle (Fig. 4e). Notably, we also found the smallest region we could visually pattern was 0.8 mm^2^, which gave us a working estimate of the resolution of the approach (Fig. 4f).

We then set out to understand the optimal level of light-sensor expression in *K. rhaeticus* to maximize the dynamic range between light and dark states. We studied this by measuring blue-light responses from the *K. rhaeticus* Opto-T7RANP variants exposed to a range of arabinose concentrations when grown with shaking conditions in parallel in microtiter plates. In these experiments, we tested five variants of the Opto-T7RANP system first created by Baumschlager et al.^49^, which had been placed onto the *K. rhaeticus* chromosome (Extended Data Fig. 7c). After evaluating the *mCherry* expression response from exposure to blue light for 6 h, we found that only one of the integrated variants (*K. rhaeticus* Opto-T7RNAP(563-F1)-*mCherry*) showed an *mCherry* expression level above that of the baseline (*K. rhaeticus*_*p*_*T7-mCherry*)—the negative control strain containing just the target plasmid (_*p*_*T7-mCherry*)—without T7-RNA polymerase (Extended Data Fig. 7c). The maximal fold change from *K. rhaeticus* Opto-T7RNAP(563-F1)-*mCherry* was at 1% (wt/vol) arabinose and gave a 7.4× fold change in mCherry production. For the plasmid-based *K. rhaeticus*_p_Opto-T7RNAP*(563-F2)-*mCherry*, 0.1% arabinose was found to be the preferred condition for dark-to-light switch behavior, whereas 10% arabinose overloaded the circuit and gave high mCherry production in the dark, and no detectable fluorescence at all when given light (Fig. 4g).

Having demonstrated we could pattern gene expression with the Opto-T7RNAP system and light projection, we next moved to pattern eumelanin accumulation in a pellicle. Having successfully used *K. rhaeticus*_p_Opto-T7RNAP*(563-F2)-*mCherry* to pattern mCherry RFP expression, we decided to create an alternative version of this strain where the *mCherry* coding sequence was directly replaced with the *tyr1* coding sequence, creating *K. rhaeticus*_p_Opto-T7RNAP*(563-F2)-*tyr1*. However, when tested in parallel with the *mCherry* version, we found that the *tyr1* version had such a high level of background eumelanin production that it obscured visible patterning of eumelanin accumulation (Extended Data Fig. 7d). We, therefore, switched to the other Opto-T7RNAP variant that had also shown an appreciable blue-light response in the shaking condition experiment using the mCherry target gene—*K. rhaeticus* Opto-T7RNAP(563-F1)—to further test whether we could pattern eumelanin accumulation.

Ahead of testing, we designed a dynamic image to be projected and a new projection setup with a commercial projector to allow us to change the image during the exposure and thus measure the required exposure times to generate an appreciable eumelanin response (Extended Data Fig. 7e). Using *K. rhaeticus* Opto-T7RNAP(563-F1)-*tyr1*, we grew a BC pellicle in this device, and once grown, we exposed the pellicle to an 80-h projection (Fig. 4h). We then took the exposed pellicle, placed it into development buffer and incubated it at 30 °C for 48 h by which point we could observe rough patterning of eumelanin accumulation (Fig. 4i). Unfortunately, while the pellicle shows some evidence of patterning in places, a high degree of background eumelanin accumulation also made this patterning attempt hard to decipher. We could, however, determine from the dynamic patterning that at least 40 h was required to observe visible eumelanin accumulation when it is produced from *K. rhaeticus* Opto-T7RNAP(563-F1)-*tyr1*.

Finally, we assessed how the other Opto-T7RNAP variants behaved with *tyr1* as their target gene. We used a similar parallel microtiter plate approach as before but now measuring the accumulation of eumelanin per initial cell over time at OD_405_ (Fig. 4j). This revealed that *K. rhaeticus*_p_Opto-T7RNAP*(563-F2)-*tyr1* required 0.1% arabinose to work correctly, as it had when *mCherry* was the target (Fig. 4g). However, now in this condition and all others tested, a high eumelanin production rate is observed even in the absence of light induction. This finding agrees with the high background pigmentation seen in the test pellicle. Among the strains with chromosomally integrated DNA, *K. rhaeticus* Opto-T7RNAP(563-F1)-*tyr1* showed the highest eumelanin production rate in response to blue light, again requiring 0.1% arabinose to tune this. The fold change between the light and dark states for this strain was lower than that for two other variants tested, which both showed fold changes greater than ten times (Extended Data Fig. 7F). However, these strains are not ideal for pigmentation, as they have much lower eumelanin production rates. Overall, while we can show the control of eumelanin production can be regulated with blue light using the Opto-T7RNAP system, accurate patterning of eumelanin accumulation in a pellicle remains to be optimized.

Our main limitations to patterning eumelanin accumulation using Opto-T7RNAP were high levels of background pigmentation and a constrained fold change in response to blue light. These two factors severely reduced the dynamic range of the system. Other factors that could have also reduced pattern definition in the pellicle include Tyr1 enzyme or l-DOPA leaking from cells (for example via cell lysis), or *tyr1* expression leading to reduced cell growth and density in blue-light-exposed regions. Most of these factors could be addressed by decreasing background *tyr1* expression from the target plasmid, but the exact source of this background expression is currently unknown. Optimization of the arrangement of the Opto-T7RNAP genes in *K. rhaeticus* will hopefully allow us to approach the dynamic range of the system seen in *E. coli*^49^. We anticipate that improving the performance of the Opto-T7RNAP system in *K. rhaeticus*, and developing alternative target genes, will yield more advanced BC biomaterials in the near future.

## Discussion

Here we employ genetic engineering in a material-producing bacteria for the purpose of creating pigmented products. We have demonstrated that tyrosinase expression from *K. rhaeticus* is proficient to produce highly pigmented BC; that the growth of engineered *K. rhaeticus* can be scaled to produce useful quantities of pigmented BC and that this pigmentation is stable. This study demonstrates the value of using genetic engineering to design and construct strains intended to grow materials with desired properties; in this case with a chosen color grown into the material, rather than having to be added to it later by an industrial chemical dyeing process.

Our study represents only the first step in the development of melanated BC. The use of the two-step process for eumelanin production increases the amount of water required for melanated BC and this may restrict its sustainability. Potentially, this additional water usage could be reduced through the reuse and recycling of development buffer, or the addition of the buffer components directly to the growth media after pellicle production. However, the most effective approach may be to adapt eumelanin production to occur in the acidic conditions generated by *K. rhaeticus*. Such an approach will require an evaluation of the eumelanin chemical pathway to identify the sources of sensitivity to low pH and a survey of natural melanin biosynthesis mechanisms to identify acid-tolerant pathways. The identification or directed evolution of acid-tolerant tyrosinase enzymes is an especially promising avenue that may enable a future one-step process.

We are confident that the production of melanated BC can be scaled further in an industrial context. While we have demonstrated that Tyr1 functions inside *K. rhaeticus*, many other *Komagataeibacter* species are used in industrial BC production and the culturing conditions to maximize yields from each species are quite varied. Therefore, to increase the flexibility of our study, the genetic engineering of *tyr1* expression should next be demonstrated in other *Komagataeibacter* species. At the industrial level, it will be important that melanated BC is subject to more rigorous testing of industry standards for color fastness, such as resistance to rubbing and fading in UV and visible light. Additionally, the sustainability credentials of melanated BC should also be assessed through a full life-cycle analysis. Melanated BC will also face the challenges that already limit the use of BC as a nonwoven textile. Chiefly, the high hydrophilicity of BC mandates significant waterproofing during the textile processing stage. Various hydrophobic coatings have been used for waterproofing cellulose-based materials with synthetic or natural compounds, and this technique has been shown to decrease the hydrophilicity of BC by coating with wax and oil from plant sources^51^. Potentially, however, this process could also be reconsidered by genetic engineering of *Komagataeibacter*, either to alter the grown BC structure, or through the biosynthesis of a layer of hydrophobic compounds. Additionally, the sterilization of BC materials, especially in the case of high-pressure steam sterilization, carries significant energy requirements that will need to be considered.

Finally, there are additional avenues to explore the genetic engineering approach, demonstrated here, to produce other pigment molecules from *K. rhaeticus*. The l-DOPA produced by tyrosinase can, in the presence of cysteine, be shifted to the formation of the red pigment, pheomelanin, and further study here would expand the possible shade range of melanated BC materials^52^. The biosynthesis of other insoluble pigment molecules could also be pursued. The most obvious is the production of indigoid dyes, such as indigo and tyrian purple, whose biosynthesis has already been demonstrated in *E. coli*^53,54^. Indeed, the engineered biosynthesis of only a small range of pigments could deliver a supernumerary range of BC shades, due to the potential of mixing pigment production at the genetic level and through the coculturing of different pigment-producing strains.

## Methods

### *K. rhaeticus* culture conditions and culturing approaches

Two culture media were used in this study to culture *K. rhaeticus*. HS-glucose media (2% glucose, 10 g l^−1^ yeast extract, 10 g l^−1^ peptone, 2.7 g l^−1^ Na_2_HPO_4_ and 1.3 g l^−1^ citric acid, pH 5.6–5.8) and coconut water media (coconut water (Vita Coco), 0.05% (vol/vol) acetic acid). Coconut water media was sterilized by filtration, except in situations where more than 1 l was required. In those situations, media supplements were sterilized separately and combined with coconut water, which had been opened and decanted out with aseptic technique, in the culturing container.

When *K. rhaeticus* was cultured on solid media, HS-glucose media was always used and supplemented with 1.5% agar. *K. rhaeticus* liquid cultures fell into the following two separate approaches: shaking cultures and stationary cultures. In shaking cultures, the media in use was supplemented with 2% (vol/vol) cellulase (Sigma-Aldrich, C2730) to allow for turbid growth without clumping. In stationary culture, where the goal is pellicle formation, media would be supplemented with 1% (vol/vol) ethanol to enhance pellicle production. In both approaches, where antibiotics were required for plasmid maintenance, media was supplemented with 340 μg ml^−1^ chloramphenicol or 200 μg ml^−1^ spectinomycin.

To facilitate consistency when inoculating multiple pellicles, *K. rhaeticus* cells would be grown in shaking growth conditions until turbid, normalized in OD_600_ across samples, pelleted by centrifugation and washed in the subsequent media to remove cellulase. The washed cells were used as a preculture and added, at a ratio of 1:25, into the culturing container and left in stationary conditions at 30 °C to form pellicles. In the case of forming large pellicles consistently (>25 cm^2^), a glycerol aliquot approach was used. The *K. rhaeticus* strain of interest would be grown, shaking at 30 °C in 100 ml of HS-glucose media until it reached an OD_600_ of ~0.6 to 1. At this point, the cells would be pelleted by centrifugation, washed in HS-glucose media, before being pelleted again and resuspended in 10 ml of HS-glucose media containing 25% glycerol. The resuspended cells would be separated into 1 ml aliquots and stored at −80 °C until use. When used, an aliquot would be thawed and added to the media in the final culturing container.

### Molecular biology and strain construction

DNA parts and plasmids used in this study are listed in the supplementary materials. *E. coli* Turbo (NEB) cells were used for plasmid construction. The *tyr1* DNA sequence was ordered from Twist Bioscience, with compatible 3′ and 5′ overhangs for entry into the KTK via Golden Gate Cloning. Constitutive tyrosinase constructs were built using the KTK. The procedures and protocols for working with the KTK are described in ref. ^26^. Plasmids containing the various versions of the Opto-T7RNAP system were kindly sent to us by A. Baumschlager and M. Khammash from ETH Zürich. Due to the presence of multiple KTK-incompatible restriction sites in the T7-Opto coding sequences, Gibson cloning was used to build both the _p_Opto-T7RNAP*(563-F2)*-target* plasmid and the five _p_Opto-T7RNAP suicide plasmids for genomic integration. The primers for Gibson cloning are listed in the supplementary materials.

*K. rhaeticus* electrocompetent cells were prepared as in ref. ^24^*. K. rhaeticus* cells were transformed using electroporation and selected for HS-glucose agar plates containing either 340 μg ml^−1^ chloramphenicol or 500 μg ml^−1^ spectinomycin, depending on the plasmid selection marker in use. Note, here a higher concentration of spectinomycin is used during normal culturing. Genetic constructs that were integrated into the chromosome of *K. rhaeticus* were done so by homologous recombination using a pUC19 suicide plasmid, as described in ref. ^26^.

### Melanated pellicle production

Melanated pellicles were produced using a two-step approach. First, a *tyr1* expression strain would be inoculated into a sterile culture container. Typically, 24-well deep well plates (Axygen) were used to make small pellicles. Each well contained 5 ml of growth media and was inoculated at a ratio of 1:25 with preculture. Growth media was enriched with 0.5 g l^−1^ l-tyrosine and 10 μM CuSO_4_ to promote the highest eumelanin production. Once the pellicles had reached the desired thickness, they were collected, placed in a bath of sterile dH_2_O and washed for 1 min by gently shaking by hand. The washed pellicles are then passed into a bath of eumelanin development buffer. A large ratio of buffer to pellicle was used, that is, one pellicle in 25 ml of buffer in a 50-ml falcon tube; this was to prevent the overwhelming of the buffer by remaining acid in the pellicle. The pellicle would be allowed to produce eumelanin at >30 °C in shaking conditions over 24 h.

### Large melanated pellicle production

To produce the melanated pellicle used to make the wallet, a 200 × 300 Eurobox container was sterilized and filled with 3 l of coconut water media supplemented with 0.5 g l^−1^ l-tyrosine, 10 μM CuSO_4_ and 1% ethanol. The media was inoculated with a 1 ml *K. rhaeticus ctyr1* glycerol aliquot and covered in a paper towel before being placed into a stationary incubator set to 30 °C. After 10 days of growth, the pellicle was collected, washed briefly in dH_2_O before being placed in a 300 × 400 mm Eurobox containing 2 l of concentrated eumelanin development buffer (10× PBS). The development container was then placed into a shaking incubator set to 45 °C and allowed to produce eumelanin over 2 days, at which point the cellulose had become completely black. The melanated cellulose was then washed again to remove excess eumelanin development buffer before being autoclaved. To make the material pliable after drying, the cellulose sheet was left in a 5% glycerol solution. This glycerol process may improve the strength of dried BC by maintaining some of the properties of wet BC, by preventing hornification^55^. The sample was then pressed to remove bulk water and air-dried for 24 h. This process typically leads to around a 98% reduction in mass due to the removal of water.

To produce the melanated pellicle used to make the shoe, a custom-shaped vessel, containing an apparatus that held a network of tightly strung yarn, was sterilized and filled with 2 l of coconut water media supplemented with 0.5 g l^−1^ l-tyrosine, 10 μM CuSO_4_, 340 μg ml^−1^ chloramphenicol and 1% ethanol. The media was inoculated with a ~500 ml precultured *K. rhaeticus ptyr1* pellicle. To accommodate the fed-batch procedure and unique vessel size necessary to incorporate the yarn apparatus, the culture was left to grow at room temperature in stationary conditions, until a thin pellicle had formed. At this point, fresh coconut water media supplemented with 0.5 g l^−1^ l-tyrosine, 10 μM CuSO_4_, 340 μg ml^−1^ chloramphenicol and 1% ethanol was added, to raise the pellicle to just below the level of the tensed yarn. After a longer growth period of 2 weeks due to lower temperature, the media was drained and replaced with concentrated eumelanin development buffer (10× PBS). The full container was placed into a shaking incubator set to 30 rpm, and developed at 30 °C for 1 day, at which point the pellicle had become completely black. The vessel was then drained of eumelanin development buffer, replaced with 70% ethanol and left overnight to sterilize. The ethanol was replaced with a 5% glycerol solution before the melanated cellulose was removed from the apparatus and wrapped around a shoe-shaped mold (last) to air-dry at 45 °C for 24 h. Once air-dried, the shoe upper and last were placed onto a sole and photographed.

### Eumelanin production assay

The eumelanin production assay uses a 384-square-well microtiter plate as a reaction plate. An OT-2 liquid handling robot (Opentrons) was used to prepare these reaction plates for the assay. Development buffer was placed into a deep well plate, from which 40 μl was transferred to each well in the reaction plate using an eight-channel 300 μl OT-2 Gen2 pipette. The reaction plate was kept at 4 °C to slow eumelanin production during plate preparation using the OT-2 thermo-module. Cells and supernatant potentially containing tyrosinase were placed into a 96-well plate. Cells were mixed in one round of aspiration using an eight-channel 20 μl OT-2 Gen2 pipette before 10 μl of cells were transferred into each well of the 384-well plate. Once full, the reaction plate was centrifuged for 10 s to draw liquid to the bottom of the wells before being sealed with a Breath-Easy sealing membrane. The reaction plate was placed into a plate reader and heated to 45 °C to accelerate eumelanin production and prevent potential cell growth from affecting optical density readings. To measure cell density in the reaction plate, an initial measurement at OD_600_ is taken, after which OD_405_ measurements are taken every 10 min for 12 h, while the plate is shaken at high speed.

### Eumelanin production assay (supernatant)

*K. rhaeticus* *ptyr1*, *K.rhaeticus tyr1*, and wild-type *K. rhaeticus* starter cultures were grown in 3 ml of HS-glucose media, with 2% cellulase, 0.5 g l^−1^ tyrosine, 10 μM CuSO_4_ and, if appropriate, 340 μg ml^−1^ chloramphenicol, in shaking conditions for 24 h. The cultures were normalized for OD_600_ and inoculated into shaking flasks containing 25 ml of the same prior media for 48 h. At this point, the cells were pelleted by centrifugation and the supernatant was transferred to a separate container on ice. The supernatant was sterilized using a 0.2-μm filter and the pH was adjusted to pH 7 by 1 M NaOH titration. The cell pellets were resuspended in eumelanin development buffer and 10 μl of the resulting mixture was placed into a 384-well plate alongside pH-adjusted supernatant samples and pH-adjusted cell cultures. Once full, the reaction plate was centrifuged for 10 s to draw liquid to the bottom of the wells before being sealed with a Breath-Easy sealing membrane. Assay plate was run using the same protocol as used in the Eumelanin production assay.

### Wettability experiments

*K. rhaeticus ptyr1* was inoculated into a 24-well deep well plate, with each well containing 5 ml of HS-glucose media, with 0.5 g l^−1^ tyrosine, 10 μM CuSO_4_ and 340 μg ml^−1^ chloramphenicol. After incubating at 30 °C for 7 days, pellicles were collected. Eumelanin production was initiated by placing the collected pellicles into eumelanin development buffer. A set of pellicles were held back from eumelanin production and placed into an acetate buffer containing 0.5 g l^−1^ tyrosine and 10 μM CuSO_4_ at pH 3.6 to act as a negative control. Melanated and unmelanated pellicles were sterilized by placing them in 70% ethanol overnight. Pellicles were then washed in distilled water to remove leftover ethanol and salt. Pellicles were then dried flat using a heated press set to 120 °C and 1 ton of pressure. This process on average leads to a 98% reduction in mass of the pellicle. To facilitate this drying and prevent the pellicles from sticking to the press, pellicles were sandwiched between three layers of filter paper. Wettability tests were conducted using a KRUSS EasyDrop with 1 μl of water. Each contact angle measurement was derived from the average contact angle from ten back-to-back water drop images taken within 10 s of drop contact with the pellicle surface.

### Tensile strength experiments

*K. rhaeticus ptyr1* was inoculated into 15-cm square Petri dishes containing 50 ml of HS-glucose media, with 0.5 g l^−1^ tyrosine, 10 μM CuSO_4_ and 340 μg ml^−1^ chloramphenicol. After incubating at 30 °C for 7 days, pellicles were collected and cut into half. One half was placed into an eumelanin development buffer to initiate eumelanin production and the other half into an acetate buffer containing 0.5 g l^−1^ tyrosine and 10 μM CuSO_4_ at pH 3.6 to prevent eumelanin production. After 24 h of shaking at 30 °C, melanated and unmelanated pellicles were removed from their respective buffers and sterilized in a 70% ethanol solution overnight. Pellicles were then washed in distilled water to remove ethanol and salts left over from the eumelanin development processes. Pellicles were then dried flat using a heated press set to 120 °C and 1 ton of pressure. This process on average leads to a 98% reduction in mass of the pellicle. The 35 -mm-long dog-bone test specimens were cut out of the dried cellulose using a Zwick ZCP 020 manual cutting press. Pellicle specimen ends reinforced with a card using Everbuild Stick 2 superglue. Dots were marked on the surface of each specimen for the optical measurement of displacement. A silver pen was used to dot melanated cellulose to generate the necessary contrast for optical measurement of displacement. Tensile tests were conducted with a Deben Microtest Tensile Stage using a load cell of 200N and cross-head speed of 0.5 mm min^−1^.

### Scanning electron microscopy

The unmelanated pellicle was prepared by placing it into an acidic acetate buffer at pH 3.6, which prevented eumelanin synthesis and incubated in identical conditions to the melanated pellicle in the eumelanin development buffer bath. Melanated and unmelanated pellicles were prepared for SEM through the following steps. Unsterilized pellicles were placed in a 20% ethanol solution and shaken gently for 1 h before being removed and placed into a 40% ethanol solution and shaken gently. This process was repeated for 60%, 80% and 100% ethanol solutions to ensure the maximum replacement of water with ethanol from the cellulose matrix. Pellicles were then flash-frozen in liquid nitrogen and freeze-dried until completely dry. The fully dried pellicles were then fixed on aluminum studs, sputter coated with gold and imaged at 5 kV with a Zeiss Auriga Gemini FEG FIB-SEM.

### Light microscopy

*K. rhaeticus ptyr1* and *K. rhaeticus ctyr1* were separately inoculated into 3 ml of HS-glucose media containing 2% (vol/vol) cellulase and 340 μg ml^−1^ chloramphenicol and grown shaking at 30 °C until turbid. The turbid cultures were then pelleted by centrifugation, washed with 1 ml PBS and split into two separate 1.5 ml centrifuge tubes. The cells were then pelleted again. One pellet was resuspended with 500 μl eumelanin development buffer to initiate eumelanin production and the other pellet was resuspended with 500 μl PBS to keep the cells unmelantated. The cells were incubated over 24 h at 30 °C by which point the tube containing the cells in eumelanin development buffer had turned black. To prepare the microscope slides, 1 μl of melanated and unmelanated cells were placed on separate 1% agarose pads and imaged on a Nikon Ti-EX1 invert microscope with a ×40 objective lens. Cells were imaged in bright field with no phase contrast to accurately represent the shade of the cells.

### Pellicle cross-sections

*K. rhaeticus* WT*, K. rhaeticus ptyr1* and *K. rhaeticus ctyr1* were inoculated into two-well deep well plates containing 50 ml of HS-glucose media, with 0.5 g l^−1^ tyrosine, 10 μM CuSO_4_ and 340 μg ml^−1^ chloramphenicol. After 10 days of incubation at 30 °C, pellicles were collected and placed into eumelanin development buffer. After 24 h, pellicles were sterilized through autoclaving. Pellicles were then placed in a −20 °C freezer for 24 h to minimize compression during sectioning. The frozen pellicles were sectioned by hand using a Leica DB80LX blade and imaged using a macro lens (Leica) on an RS Pro lightbox.

### Color resistance to water spotting

*K. rhaeticus* *ptyr1* and *K. rhaeticus* *ctyr1* were inoculated into 12.5 × 16.5 cm^2^ two-well glass container with 200 ml of HS-glucose media with 0.5 g l^−1^ tyrosine, 10 μΜ CuSO4 and 340 μg ml^−1^ chloramphenicol. After incubation for 7 days at 30 °C, pellicles were collected. Eumelanin production was initiated by placing the pellicles into eumelanin development buffer. After 24 h of shaking at 30 °C, pellicles were removed from the buffer and sterilized in 70% ethanol solution overnight. Pellicles were then washed in distilled water to remove ethanol and leftover salts. To make the material pliable after drying, replicate pellicles were placed in 0% or 5% glycerol solution overnight. Pellicles were then dried flat using a heated press set to 120 °C and 1 ton of pressure. To facilitate this drying and prevent the pellicles from sticking to the press, pellicles were sandwiched between three layers of filter paper. Water spotting tests were adapted from ISO 105-E07:2010 standard. Eumelanated pellicles were secured onto an RS Pro lightbox, and 50 μl of distilled water was spotted onto each sample in triplicate. Pellicles were imaged before, immediately after and 16 h after water spotting and assessed for color change.

### Patterning *mCherry* expression in a *K. rhaeticus*_p_Opto-T7RNAP*(563-F2)-*mCherry* pellicle

A custom projection rig was built to project light onto the growing pellicle (Extended Data Fig. 7b). This held an acetate transparency that contained various components that would test the quality of the patterning in the pellicle. The image transparency was designed in Adobe Illustrator and printed on an HP LaserJet 500 MFP M570. Four acetate transparencies were stacked atop each other to form the final transparency. This was then sealed between glass slides and secured to the upper laboratory loop clamp. The pellicle container was sterilized and filled with 500 ml of HS-glucose media, containing 0.1% (wt/vol) arabinose, 1% (vol/vol) ethanol and 170 μg ml^−1^ chloramphenicol. The media was then inoculated with a 1-ml *K. rhaeticus*_p_Opto-T7RNAP*(563-F2)-*mCherry* glycerol aliquot, and a glass lid was placed on top of the container. This glass lid was warmed before placement to prevent condensation forming on it and distorting the projection. The LED lamp was then turned on, and the lens shuttered with a piece of black card. After 3 days at ~30 °C, a thin pellicle had formed. The lens was uncovered and the image from the transparency focused on the pellicle. Once the pellicle had been exposed to the projected image for 3 days, it was collected and scanned using a FLA-5000 fluorescence scanner (Fujifilm). Image analysis was conducted using the OpenCV Python library.

### Patterning *tyr1* expression in a *K. rhaeticus* Opto-T7RNAP(563-F1)-*tyr1* pellicle

A custom rig using a commercial LED projector (ViewSonic M1) was built to project light onto the growing pellicle (Extended Data Fig. 7e). The rig was draped with blackout fabric to remove outside light. A time-lapse image was designed in Adobe Illustrator to test how long a given pellicle would need to be exposed to light before an identifiable change in pigmentation could be observed. In this image, blue is represented by an RGB value of (0, 0, 255), cyan by (0, 255, 255), white by (255, 255, 255) and black by (0, 0, 0) (Fig. 4h). The pellicle container was sterilized and filled with 1 l of coconut water media, containing 1% (wt/vol) arabinose, 0.5 g l^−1^ l-tyrosine, 10 μM CuSO_4_, 1% (vol/vol) ethanol and 200 μg ml^−1^ spectinomycin. The media was then inoculated with a 1-ml *K. rhaeticus* Opto-T7RNAP(563-F1)-t*yr1* glycerol aliquot and the culture container was covered with foil. While this version of the optogenetic rig did contain a heater, in practice, we found this was only effective at heating the growth area by 1–2 °C above room temperature. After 8 days at near room temperature (~20 °C), a thin pellicle had formed. The foil was then removed, the projector focused on the surface of the pellicle and the 80-h video started. After 80 h, the pellicle was collected and placed into a 300 × 400 mm Eurobox containing 2 l of concentrated eumelanin development buffer and left to develop in stationary conditions at 30 °C until a discernible pattern could be identified. The pellicle was then washed in dH_2_O to remove eumelanin that had not accumulated within the pellicle. Densitometry scans of the pellicle were taken using an Amersham Typhoon scanner (GE) and set to the digi-blue digitalization setting.

### Characterizing *mCherry* expressing optogenetic strains

*K. rhaeticus* Opto-T7RNAP strains carrying the _*p*_*T7-mCherry* plasmid and *K. rhaeticus* pOpto-T7RNAP*(563-F2)-*mCherry* were cultured, in darkness, shaking in 3 ml of HS-glucose media with 2% cellulase, containing either spectinomycin at 200 μg ml^−1^ or chloramphenicol at 340 μg ml^−1^ depending on the plasmid. When all cultures had become turbid, the OD_600_ was measured and cultures were all either diluted or concentrated to an OD_600_ of 1, before being inoculated (a ratio of 1:10) into a 96-well deep well plate containing 270 μl HS-glucose media with 2% cellulase and either 0, 1, 10 or 100 mg ml^−1^ of arabinose. Where appropriate, spectinomycin at 200 μg ml^−1^ and chloramphenicol at 340 μg ml^−1^ were added to the wells. After 18 h of shaking growth at 30 °C in darkness, cells were split across two clear 96-well plates, diluted 1:2 into fresh media with a matching arabinose concentration. One plate was placed onto a shaker under a blue LED flood light and the other plate was wrapped in foil and placed on the same shaker. Both plates were sealed with a Breath-Easy sealing membrane. After 6 h in the two lighting conditions at 30 °C and fast shaking, the cells were placed into a plate reader, and red fluorescence in each well was measured using ex of 590 nm and em of 645 nm as well as cell density at OD_600_.

### Characterizing *tyr1* expressing optogenetic strains

The Opto-T7RNAP *K. rhaeticus* strains carrying the _*p*_*T7-tyr1* plasmid and *K. rhaeticus* pOpto-T7RNAP(563-F1)-*tyr* were cultured in the same manner as the mCherry strains—with the exception that the HS-glucose was supplemented with 0.5 g l^−1^ tyrosine and 10 μM CuSO_4_. The approach to exposing the cells to blue light was also the same as the mCherry strains, except, after 6 h of exposure time, the two plates were entered into the eumelanin production assay procedure. The two plates were placed onto the OT-2 deck and samples from both plates were mixed with eumelanin development buffer in a 384-well reaction plate. Each well in the two 96-well plates was sampled twice in the 384 reaction plate to give two technical replicates for each well. These two replicates were then averaged during analysis.

### Reporting summary

Further information on research design is available in the Nature Portfolio Reporting Summary linked to this article.

## Online content

Any methods, additional references, Nature Portfolio reporting summaries, source data, extended data, supplementary information, acknowledgements, peer review information; details of author contributions and competing interests; and statements of data and code availability are available at 10.1038/s41587-024-02194-3.

## Supplementary information

Supplementary information Supplementary Figs. 1–5, Tables 1–3 and Supplementary Data 1 and 2.

Reporting summary

Supplementary Video 1 Video of a 30 × 20 c, sterile, pressed and dried melanated BC sheet showing its texture and flexibility.

## Source data

Source Data Fig. 1,3,4 Statistical source data.

Source Data Fig. 4 Unformatted pellicle images and scans.

## Data Availability

Source data for eumelanin production rates (Fig. 1e), material property characterization (Fig. 3) and blue-light-induced gene expression (Fig. 4) are available to download. Raw images from the optogenetics experiments (Fig. 4) are also provided as Supplementary Data. Larger file size raw images, and light and electron microscopy images of samples will be shared on request. Source data are provided with this paper.
