# Supplementary material for: Self-pigmenting textiles grown from cellulose-producing bacteria with engineered tyrosinase expression
Source: Nat Biotechnol. 2024 Apr 2;43(3):345–54. doi: 10.1038/s41587-024-02194-3 (PMC11919691; doi:10.1038/s41587-024-02194-3)
Supplement: Supplementary file 1 — Supplementary Figs. 1–5, Tables 1–3 and Supplementary Data 1 and 2. [file 41587_2024_2194_MOESM1_ESM.pdf]

nature biotechnology

Article
https://doi.org/10.1038/s41587-024-02194-3

Self-pigmenting textiles grown from cellulose-producing bacteria with engineered tyrosinase expression

In the format provided by the
authors and unedited

Supplementary table 1. Strains used in this study

**Strains**	**Description**	**Reference**
K. rhaeticus iGEM	Strain of bacterial cellulose producing bacteria isolated from a kombucha tea SCOBY	Florea *et al.*
K. rhaeticus ptyr1	Constitutive production of Tyr1 protein. Possess chloramphenicol resistance	This study
K. rhaeticus ctyr1	Constitutive production of Tyr1 protein from integrated tyr1 gene at the *arsH* locus. Possess chloramphenicol resistance.	This study
K. rhaeticus $_{p}$Opto-T7RNAP*(563-F2)-mCherry	Constitutive AraC production confers arabinose sensitivity. Expression of both halves of the Opto-T7RNAP*(563-F1) protein are under pBad promoter control, and are upregulated in an increase of arabinose concentration. Blue light sensitivity in presence of arabinose leads to expression of mCherry under the T7 promoter. Possess chloramphenicol resistance.	This study
K. rhaeticus Opto-T7RNAP*(563-F1)-*mCherry*	Constitutive AraC production confers arabinose sensitivity. Expression of both halves of the Opto-T7RNAP*(563-F1) protein are under pBad promoter control, and are upregulated in an increase of arabinose concentration. Blue light sensitivity in presence of arabinose leads to expression of mCherry under the T7 promoter. Possess chloramphenicol and spectinomycin resistance.	This study
K. rhaeticus Opto-T7RNAP*(563-F2)-*mCherry*	Constitutive AraC production confers arabinose sensitivity. Expression of both halves of the Opto-T7RNAP*(563-F2) protein are under pBad promoter control, and are upregulated in an increase of arabinose concentration. Blue light sensitivity in presence of arabinose leads to expression of mCherry under the T7 promoter. Possess chloramphenicol and spectinomycin resistance.	This study
K. rhaeticus Opto-T7RNAP*(69)-*mCherry*	Constitutive AraC production confers arabinose sensitivity. Expression of both halves of the Opto-T7RNAP*(69) protein are under pBad promoter control, and are upregulated in an increase of arabinose concentration. Blue light sensitivity in presence of arabinose leads to expression of mCherry under the T7 promoter. Possess chloramphenicol and spectinomycin resistance.	This study
K. rhaeticus Opto-T7RNAP*(563)-*mCherry*	Constitutive AraC production confers arabinose sensitivity. Both halves of the Opto-T7RNAP*(563) gene are under pBad promoter control, and are upregulated in an increase of arabinose concentration. Blue light sensitivity in presence of arabinose leads to expression of mCherry under the T7 promoter. Possess chloramphenicol and spectinomycin resistance.	This study
K. rhaeticus Opto-T7RNAP(563-F1)-*mCherry*	Constitutive AraC production confers arabinose sensitivity. Both halves of the Opto-T7RNAP(563-F1) gene are under pBad promoter control, and are upregulated in an increase of arabinose concentration. Blue light sensitivity in presence of arabinose leads to expression of mCherry under the T7 promoter. Possess chloramphenicol and spectinomycin resistance.	This study

K. rhaeticus $_p$*T7-mCherry*	*mCherry* gene under the T7 promoter control. Does not contain a T7 polymerase gene. Possess spectinomycin resistance.	This study
K. rhaeticus $_p$Opto-T7RNAP*(563-F2)-*tyr1*	Constitutive AraC production confers arabinose sensitivity. Expression of both halves of the Opto-T7RNAP*(563-F1) protein are under pBad promoter control, and are upregulated in an increase of arabinose concentration. Blue light sensitivity in presence of arabinose leads to expression of Tyr1 under the T7 promoter. Possess chloramphenicol resistance.	This study
K. rhaeticus Opto-T7RNAP*(563-F1)-*tyr1*	Constitutive AraC production confers arabinose sensitivity. Expression of both halves of the Opto-T7RNAP*(563-F1) protein are under pBad promoter control, and are upregulated in an increase of arabinose concentration. Blue light sensitivity in presence of arabinose leads to expression of Tyr1 under the T7 promoter. Possess chloramphenicol and spectinomycin resistance.	This study
K. rhaeticus Opto-T7RNAP*(563-F2)-*tyr1*	Constitutive AraC production confers arabinose sensitivity. Expression of both halves of the Opto-T7RNAP*(563-F2) protein are under pBad promoter control, and are upregulated in an increase of arabinose concentration. Blue light sensitivity in presence of arabinose leads to expression of Tyr1 under the T7 promoter. Possess chloramphenicol and spectinomycin resistance.	This study
K. rhaeticus Opto-T7RNAP*(69)-*tyr1*	Constitutive AraC production confers arabinose sensitivity. Expression of both halves of the Opto-T7RNAP*(69) protein are under pBad promoter control, and are upregulated in an increase of arabinose concentration. Blue light sensitivity in presence of arabinose leads to expression of Tyr1 under the T7 promoter. Possess chloramphenicol and spectinomycin resistance.	This study
K. rhaeticus Opto-T7RNAP*(563)-*tyr1*	Constitutive AraC production confers arabinose sensitivity. Both halves of the Opto-T7RNAP*(563) gene are under pBad promoter control, and are upregulated in an increase of arabinose concentration. Blue light sensitivity in presence of arabinose leads to expression of Tyr1 under the T7 promoter. Possess chloramphenicol and spectinomycin resistance.	This study
K. rhaeticus Opto-T7RNAP(563-F1)-*tyr1*	Constitutive AraC production confers arabinose sensitivity. Both halves of the Opto-T7RNAP(563-F1) gene are under pBad promoter control, and are upregulated in an increase of arabinose concentration. Blue light sensitivity in presence of arabinose leads to expression of Tyr1 under the T7 promoter. Possess chloramphenicol and spectinomycin resistance.	This study
K. rhaeticus $_p$*T7-tyr1*	*tyr1* gene under the T7 promoter control. Does not contain a T7 polymerase gene. Possess spectinomycin resistance.	This study

Supplementary table 2. Plasmids used in this study

**Plasmid name**	**Description and construction**	**Reference**
$_p$Tyr1	Plasmid constructed with KTK. pBBR1 origin of replication and chloramphenicol resistance cassette.	This study

	J23104-B0034-Tyr1-L321P00	
$_{p}$Tyr1_IV	Plasmid constructed with KTK. pUC19 origin of replication and ampicillin resistance cassette. J23104-B0034-Tyr1-L321P00	This study
$_{p}$T7-mCherry	Plasmid constructed with Gibson cloning. pBBR1 origin of replication and spectinomycin resistance cassette. The *mCherry* coding sequence, RBS, terminator, and promoter were taken from pAB50 from Baumschlager *et al.*	This study
$_{p}$T7-tyr1	Plasmid constructed with Gibson cloning. pBBR1 origin of replication and spectinomycin resistance cassette. Derived from $_{p}$T7-mCherry, using the same RBS, terminator, promoter but switching the mCherry CDS for the *tyr1* CDS.	This study
$_{p}$Opto-T7RNAP*(563-F2)-*mCherry*	Plasmid constructed with Gibson cloning. pBBR1 origin of replication and chloramphenicol resistance cassette. Both Opto-T7RNAP*(563-F2) genes were taken from pAB152 from baumschlager *et al.*	This study
$_{p}$Opto-T7RNAP*(563-F2)-*tyr1*	Plasmid constructed with Gibson cloning. pBBR1 origin of replication and chloramphenicol resistance cassette. Both Opto-T7RNAP*(563-F2) genes were taken from pAB152 from baumschlager *et al.*	This study
$_{p}$Opto-T7RNAP*(563-F1)_IV	Plasmid constructed with Gibson cloning. pUC19 origin of replication and Ampicillin resistance cassette. Both Opto-T7RNAP*(563-F1) genes were taken from pAB151 from baumschlager *et al.*	This study
$_{p}$Opto-T7RNAP*(563-F2)_IV	Plasmid constructed with Gibson cloning. pUC19 origin of replication and Ampicillin resistance cassette. Both Opto-T7RNAP*(563-F2) genes were taken from pAB152 from baumschlager *et al.*	This study
$_{p}$Opto-T7RNAP*(69)_IV	Plasmid constructed with Gibson cloning. pUC19 origin of replication and Ampicillin resistance cassette. Both Opto-T7RNAP*(69) genes were taken from pAB144 from baumschlager *et al.*	This study
$_{p}$Opto-T7RNAP*(563)_IV	Plasmid constructed with Gibson cloning. pUC19 origin of replication and Ampicillin resistance cassette. Both Opto-T7RNAP*(563) genes were taken from pAB150 from baumschlager *et al.*	This study
$_{p}$Opto-T7RNAP(563-F1)_IV	Plasmid constructed with Gibson cloning. pUC19 origin of replication and Ampicillin resistance cassette. Both Opto-T7RNAP(563-F1) genes were taken from pAB203 from baumschlager *et al.*	This study

Supplementary table 3. Sequences of oligonucleotides used in this study

Name	Sequence
Opto-t7_fwd	TTATTTGATGCCTTTAATTAAGAAGACGGCGACGACCCGGTAGTGATCTTATTTCATTAT
Opto-t7_rev	CGTATTACCTAGGCTACGCCGGTTCTTATGGCTCTTGTATC
Pt7-mcherry fwd	ATACAAGAGCCATAAGAACCGGCGTAGCCTAGGTAATACG
Pt7-mcherry rev	GCCTGGAGATCCTTACTCGAACTCCTCCTTTCGCTAGCAA
Arac fwd	TTGCTAGCGAAAGGAGGAGTTCGAGTAAGGATCTCCAGGC
Arac rev	ATCAACAGGAGTCCAAGACTAGTGAAGACCCAGGGCGTTCTGCCGTGATTATAGACACTT
Opto_t7_tyr1_gibson fwd	AGTCGAAGCGCAGCTCTTGAGGTACCCTCGAGTCTGGTAA
Opto_t7_tyr1_gibson rev	ACGCGGTATTTATTGCCCATATGCTTTACCTCCTCTATCG
Tyr1_opto fwd	CGATAGAGGAGGTAAAGCATATGGGCAATAAATACCGCGT
Tyr1_opto rev	TTACCAGACTCGAGGGTACCTCAAGAGCTGCGCTTCGACT
Opto-t7_intergration fwd	ACCGAAGGATCTGACGGAACGAGGTCTCTGACCCGACAAAAATACGCCCGGTAGTGATCT
Opto-t7_intergration rev	GTTTTATTTGATGCCTGGAGATCCTTACTCGATGAGGTTCTTATGGCTCTTGTATCTATC
Arac_intergration fwd	ACAGGAGTCCAAGACTAGTGGTCTCACGTTTCGGCTGCCGTGATTATAGACACTTTTGTT
Arac_intergration rev	GATGCTTCACTGATAGATACAAGAGCCATAAGAACCTCATCGAGTAAGGATCTCCAGGCA
Pt7-mcherry-target fwd	GAGTGGGTCTCCGACCTAATACGACTCACTATAGGGAGAG
Pt7-mcherry-target rev	GAGTGGGTCTCCCGTTATAGTCGACTCCTCCTTTCG
Tyr1-target fwd	CGTCTCCTCGGTCTCCTATGGGCAATAAATACCGCG
Tyr1-target rev	CGTCTCCGGTCTCAAGAATCAAGAGCTGCGCTTCG

Supplementary data 1. Tyr1 AA sequence.

MGNKYRVRKNVLHLTDTEKRDFVRTVLILKEKGIYDRYIAWHGAAGKFHTPPGSDRNAAHM
SSAFLPWHREYLLRFERDLQSINPEVTLPYWEWETDAQMQDPSQSQIWSADFMGGNGNPI
KDFIVDTGPFAAGRWTTIDEQGNPSGGLKRNFGATKEAPTLPTRDDVLNALKITQYDTPPW
DMTSQNSFRNQLEGFINGPQLHNRVHRWVGGQMGVVPTAPNDPVFFLHHANVDRIWAVW
QIIHRNQNYQPMKNGPFGQNFRDPMYPWNTTPEDVMNHRKLGYVYDIELRKSKRSS*

Supplementary data 2. *tyr1* DNA sequence.

ATGGGCAATAAATACCGCGTGCGTAAGAATGTTCTGCACCTGACAGATACCGAGAAGCG
TGACTTCGTGCGCACTGTACTGATTTTGAAAGAGAAGGGCATTTACGATCGTTACATCGC
ATGGCACGGCGCCGCGGGTAAGTTTCACACCCCGCCCGGTAGTGACCGTAACGCGGC
GCACATGTCGAGTGCGTTCTTGCCTTGGCACCGCGAATATCTGCTGCGCTTTGAGCGC
GATCTGCAATCGATTAACCCTGAGGTGACTCTGCCGTACTGGGAGTGGGAAACCGATGC

TCAAATGCAAGACCCTAGCCAGTCGCAGATCTGGAGCGCCGACTTCATGGGCGGCAAT
GGCAACCCAATTAAGGACTTCATTGTAGACACGGGCCCGTTCGCTGCCGGCCGTTGGA
CAACCATTGACGAGCAGGGTAACCCGTCAGGCGGCTTAAAGCGCAACTTCGGTGCGAC
TAAGGAAGCCCCCACCCTGCCGACGCGCGACGACGTGCTGAACGCACTTAAGATTACC
CAATACGACACCCCACCCTGGGACATGACGTCCCAGAATAGTTTCCGCAACCAACTCGA
GGGTTTCATCAATGGCCCGCAACTGCATAACCGTGTGCATCGCTGGGTCGGTGGCCAA
ATGGGTGTCGTCCCTACCGCGCCCAACGACCCGGTGTTCTTCCTGCATCATGCGAACG
TTGACCGCATCTGGGCCGTGTGGCAGATCATCCACCGCAACCAGAATTACCAACCAATG
AAGAATGGCCCGTTCGGCCAGAATTTCCGTGACCCAATGTATCCATGGAACACCACGCC
TGAGGATGTAATGAATCACCGTAAACTGGGCTATGTTTATGACATCGAGTTGCGTAAGTC
GAAGCGCAGCTCTTGA

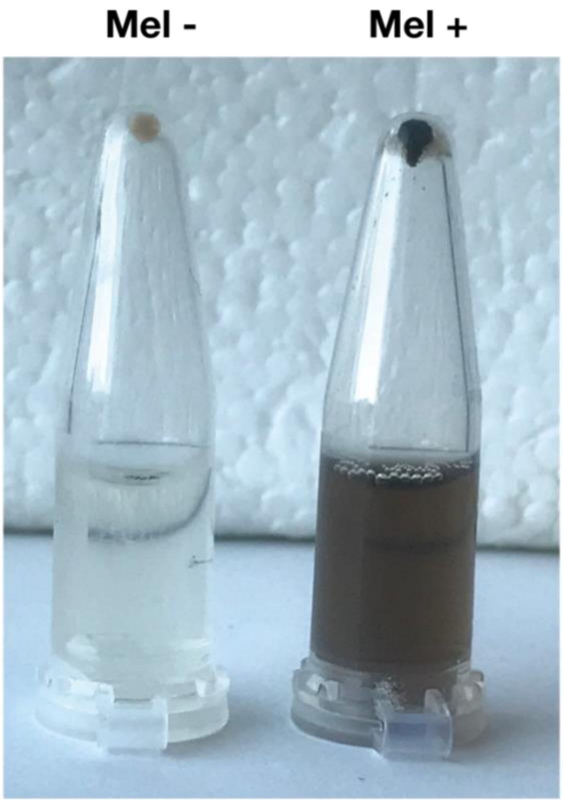

Supplementary figure 1. Pelleted unmelanated and melanated *K. rhaeticus ptyr1* cells. *K. rhaeticus ptyr1* cells were grown in HS-glucose with 340 µg/ml chloramphenicol and 2 % (v/v) cellulase. Once turbid, cells were added to HS-glucose with 0.5 g/L L-tyrosine and 10 µM $CuSO_4$ with a citrate-phosphate buffer set to either pH 5.8 (Mel -) or pH 7 (Mel +). After 24 hours of shaking incubation at 30°C, 1 mL of cells from each culture were pelleted with centrifugation. Eumelanin pigmentation can be seen in both pellet and supernatant for cells that were exposed to HS-glucose media set to pH 7.

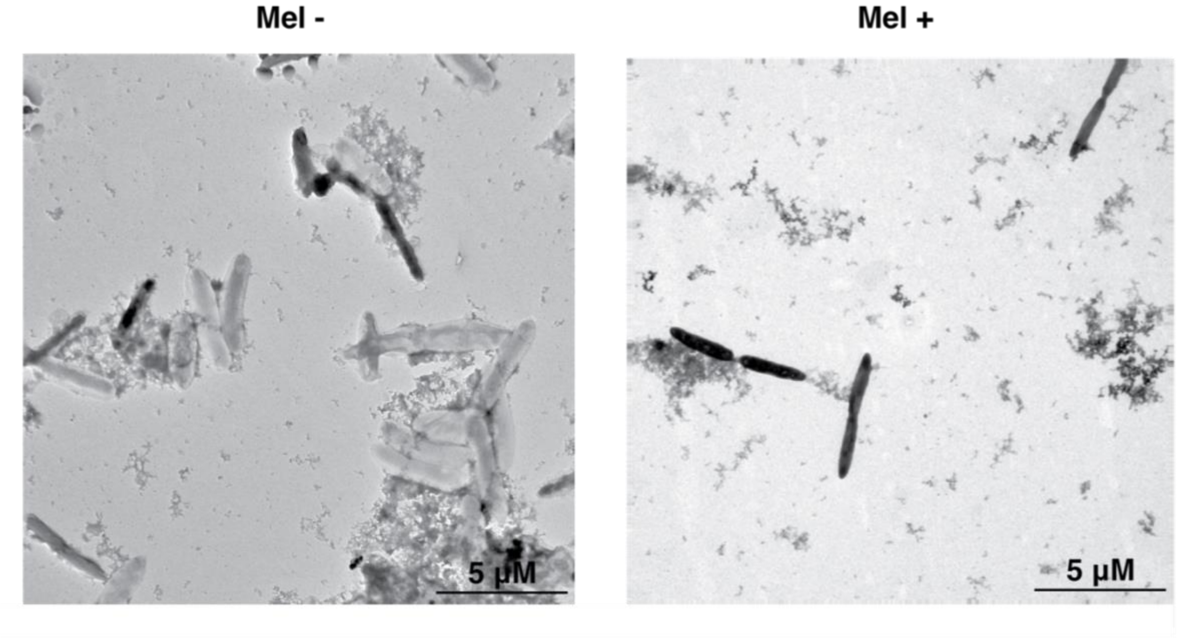

Supplementary figure 2. TEM microscopy of melanated and unmelanated *K. rhaeticus ptyr1*. Cells grown in HS-glucose media were washed in PBS, split into two separate tubes. PBS was replaced with either eumelanin development buffer or acetate buffer at pH 3.6 to produce melanated (mel +) and unmelanated cells (mel -) respectively. 2 µl samples were spotted onto 471 freshly glow discharged formvar/Carbon on 300 Mesh Nickel grids (Agar Scientific) and visualised with a FEI Tecnai G2 Spirit TWIN. Whilst cells were grown in 2% cellulase, globular matter seen in both images is likely to be incompletely digested cellulose. Images chosen are representative of 3 images of Mel - and 4 images of Mel + conditions.

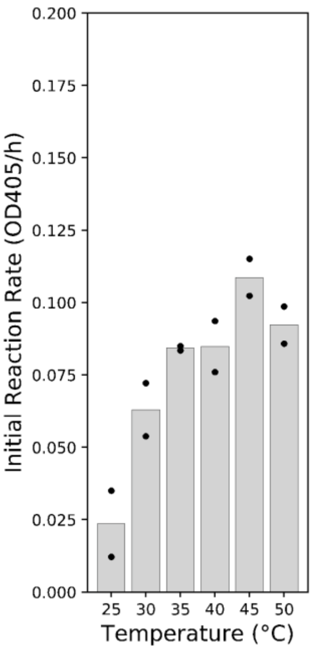

Supplementary figure 3. Initial reaction rate of eumelanin production from *K. rhaeticus ctyr1* at a range of temperatures. *K. rhaeticus ctyr1* cells were grown in HS-glucose with 0.5 g/L L-tyrosine and 10 µM $CuSO_4$ before being washed and mixed with eumelanin development buffer. Cells were then distributed across a 96 well PCR plate using 50 uL per well. The plate was then placed into a heated block with a distributed range of temperatures. Every 20 minutes, a row of sample was removed, and placed on ice. After 120 minutes had passed eumelanin accumulation was measured at OD_{405} for all temperatures and timepoints. Initial rate of reaction was calculated from the rate of eumelanin accumulation over 120 minutes. Two replicates were used for each temperature and bars show the average of these replicates.

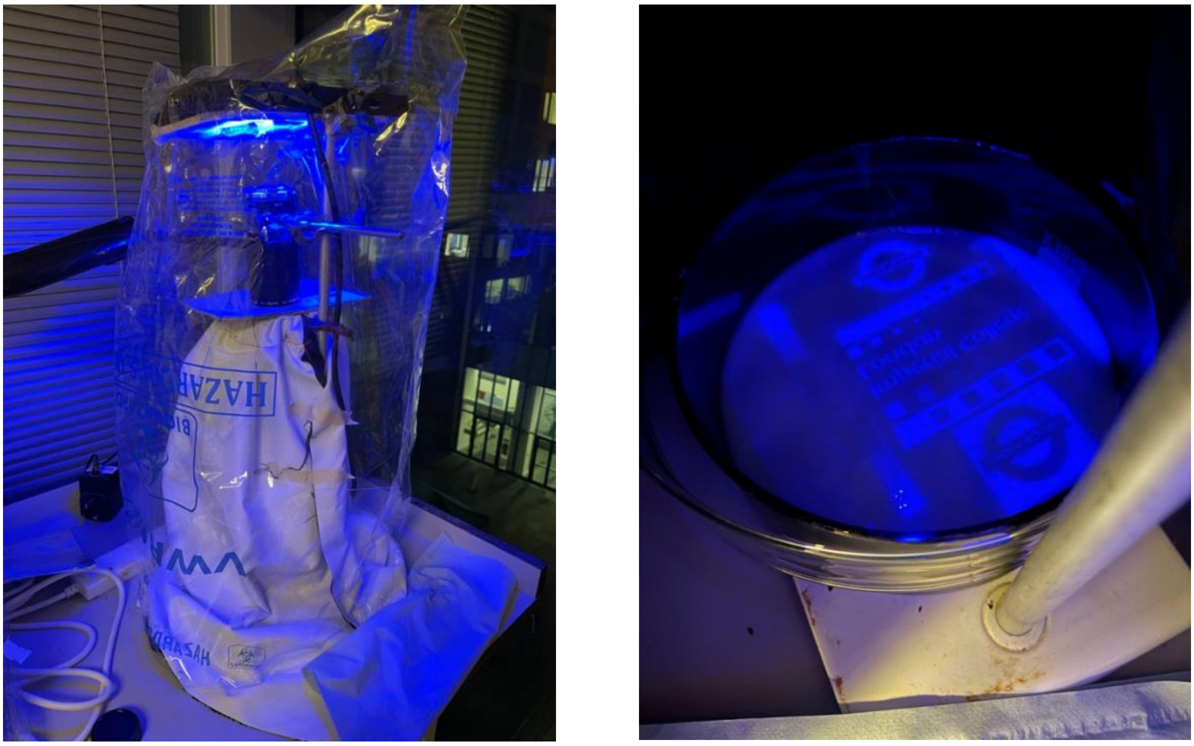

Supplementary figure 4. Photographs of optogenetic rig used to produce patterned pellicle from *K. rhaeticus* $_{p}$Opto-T7RNAP*(563-F2)-*mCherry.* Image on the right shows the full assembly used to produce the patterned pellicle. Image on the right, shows the image transparency being projected on to the pellicle.

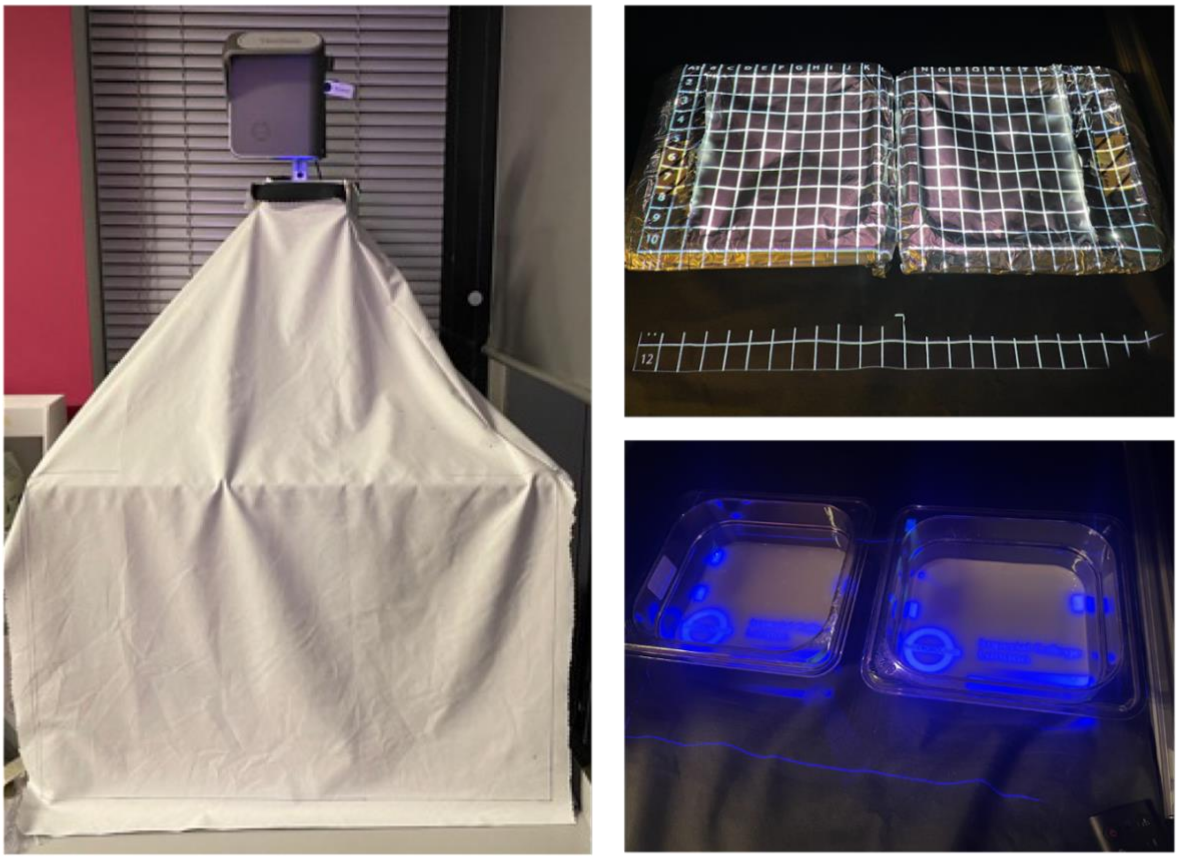

Supplementary figure 5. Photographs of optogenetic rig used to produce patterned pellicle from *K. rhaeticus* $_{\mathbf{p}}$Opto-T7RNAP(563-F1)-*tyr1*. Image on the right shows the full assembly used to produce the patterned *tyr1* pellicle. Assembly is coved in black out fabric to exclude light. Image on the top right, shows a grid being projected onto culture containers to aid in placement of image projections. Image on bottom right, shows to example images being projected onto a pair of pellicles.
