# Supplementary material for: Self-pigmenting textiles grown from cellulose-producing bacteria with engineered tyrosinase expression
Source: Nat Biotechnol. 2024 Apr 2;43(3):345–54. doi: 10.1038/s41587-024-02194-3 (PMC11919691; doi:10.1038/s41587-024-02194-3)
Supplement: Supplementary file 2 — Reporting summary [file 41587_2024_2194_MOESM2_ESM.pdf]

nature portfolio

Corresponding author(s): Tom Ellis

Last updated by author(s): Feb 26, 2024

Reporting Summary

Nature Portfolio wishes to improve the reproducibility of the work that we publish. This form provides structure for consistency and transparency in reporting. For further information on Nature Portfolio policies, see our Editorial Policies and the Editorial Policy Checklist.

Statistics

For all statistical analyses, confirm that the following items are present in the figure legend, table legend, main text, or Methods section.

n/a	Confirmed	
☐	☒	The exact sample size (*n*) for each experimental group/condition, given as a discrete number and unit of measurement
☐	☒	A statement on whether measurements were taken from distinct samples or whether the same sample was measured repeatedly
☒	☐	The statistical test(s) used AND whether they are one- or two-sided<br>*Only common tests should be described solely by name; describe more complex techniques in the Methods section.*
☒	☐	A description of all covariates tested
☒	☐	A description of any assumptions or corrections, such as tests of normality and adjustment for multiple comparisons
☐	☒	A full description of the statistical parameters including central tendency (e.g. means) or other basic estimates (e.g. regression coefficient) AND variation (e.g. standard deviation) or associated estimates of uncertainty (e.g. confidence intervals)
☒	☐	For null hypothesis testing, the test statistic (e.g. *F*, *t*, *r*) with confidence intervals, effect sizes, degrees of freedom and *P* value noted<br>*Give P values as exact values whenever suitable.*
☒	☐	For Bayesian analysis, information on the choice of priors and Markov chain Monte Carlo settings
☒	☐	For hierarchical and complex designs, identification of the appropriate level for tests and full reporting of outcomes
☒	☐	Estimates of effect sizes (e.g. Cohen's *d*, Pearson's *r*), indicating how they were calculated

Our web collection on statistics for biologists contains articles on many of the points above.

Software and code

Policy information about availability of computer code

Data collection	Pigment density measurements were taken using Amersham Typhoon with data collected using the Scanner Control Software v3.0
Data analysis	Data analysis for bar chart creation was done using Microsoft Excel version 15

For manuscripts utilizing custom algorithms or software that are central to the research but not yet described in published literature, software must be made available to editors and reviewers. We strongly encourage code deposition in a community repository (e.g. GitHub). See the Nature Portfolio guidelines for submitting code & software for further information.

Data

Policy information about availability of data

All manuscripts must include a data availability statement. This statement should provide the following information, where applicable:

- Accession codes, unique identifiers, or web links for publicly available datasets
- A description of any restrictions on data availability
- For clinical datasets or third party data, please ensure that the statement adheres to our policy

Source data for eumelanin production rates (Fig 1e), material property characterisation (Fig 3), and blue-light induced gene expression (Fig 4) are available to download. Raw images from the optogenetics experiments (Fig 4) are also provided as supplementary data. Larger file size raw images, and light and electron microscopy images of samples will be shared on request.

Research involving human participants, their data, or biological material

Policy information about studies with human participants or human data. See also policy information about sex, gender (identity/presentation), and sexual orientation and race, ethnicity and racism.

Reporting on sex and gender	N/A
Reporting on race, ethnicity, or other socially relevant groupings	N/A
Population characteristics	N/A
Recruitment	N/A
Ethics oversight	N/A

Note that full information on the approval of the study protocol must also be provided in the manuscript.

Field-specific reporting

Please select the one below that is the best fit for your research. If you are not sure, read the appropriate sections before making your selection.

☒ Life sciences ☐ Behavioural & social sciences ☐ Ecological, evolutionary & environmental sciences

For a reference copy of the document with all sections, see nature.com/documents/nr-reporting-summary-flat.pdf

Life sciences study design

All studies must disclose on these points even when the disclosure is negative.

Sample size	Experiments that created quantitative data were always at least n=3 in order to calculate mean and standard deviation. For optogenetics experiments on growing pellicles, these were considered as 'demonstration runs' (demos) and so sample size was less, only n=2 for pellicles with RFP output and n=1 for pellicles with melanin ouput.
Data exclusions	No data points were excluded when obtaining quantative data. As mentioned in the manuscript, several pellicles where we tried to pattern melanin production via optogenetic induction were failures and the images of these failed experiments were not included.
Replication	For all quantitative data in the manuscript at least 3 biological replicates were used.<br>For growth of materials, pellicle growth was highly variable, so demonstration results are shown.
Randomization	Not relevant to this study, as not experiments were designed to be assessed by statistical methods
Blinding	Not relevant to this study, as not experiments were designed to be assessed by statistical methods

Reporting for specific materials, systems and methods

We require information from authors about some types of materials, experimental systems and methods used in many studies. Here, indicate whether each material, system or method listed is relevant to your study. If you are not sure if a list item applies to your research, read the appropriate section before selecting a response.

Materials & experimental systems

n/a	Involved in the study	
☒	☐	Antibodies
☒	☐	Eukaryotic cell lines
☒	☐	Palaeontology and archaeology
☒	☐	Animals and other organisms
☒	☐	Clinical data
☒	☐	Dual use research of concern
☒	☐	Plants

Methods

n/a	Involved in the study	
☒	☐	ChIP-seq
☒	☐	Flow cytometry
☒	☐	MRI-based neuroimaging

Plants

Seed stocks	N/A
Novel plant genotypes	N/A
Authentication	N/A
