# Supplementary figures and images for: Self-pigmenting textiles grown from cellulose-producing bacteria with engineered tyrosinase expression
Source: Nat Biotechnol. 2024 Apr 2;43(3):345–54. doi: 10.1038/s41587-024-02194-3 (PMC11919691; doi:10.1038/s41587-024-02194-3)

Figure 4D

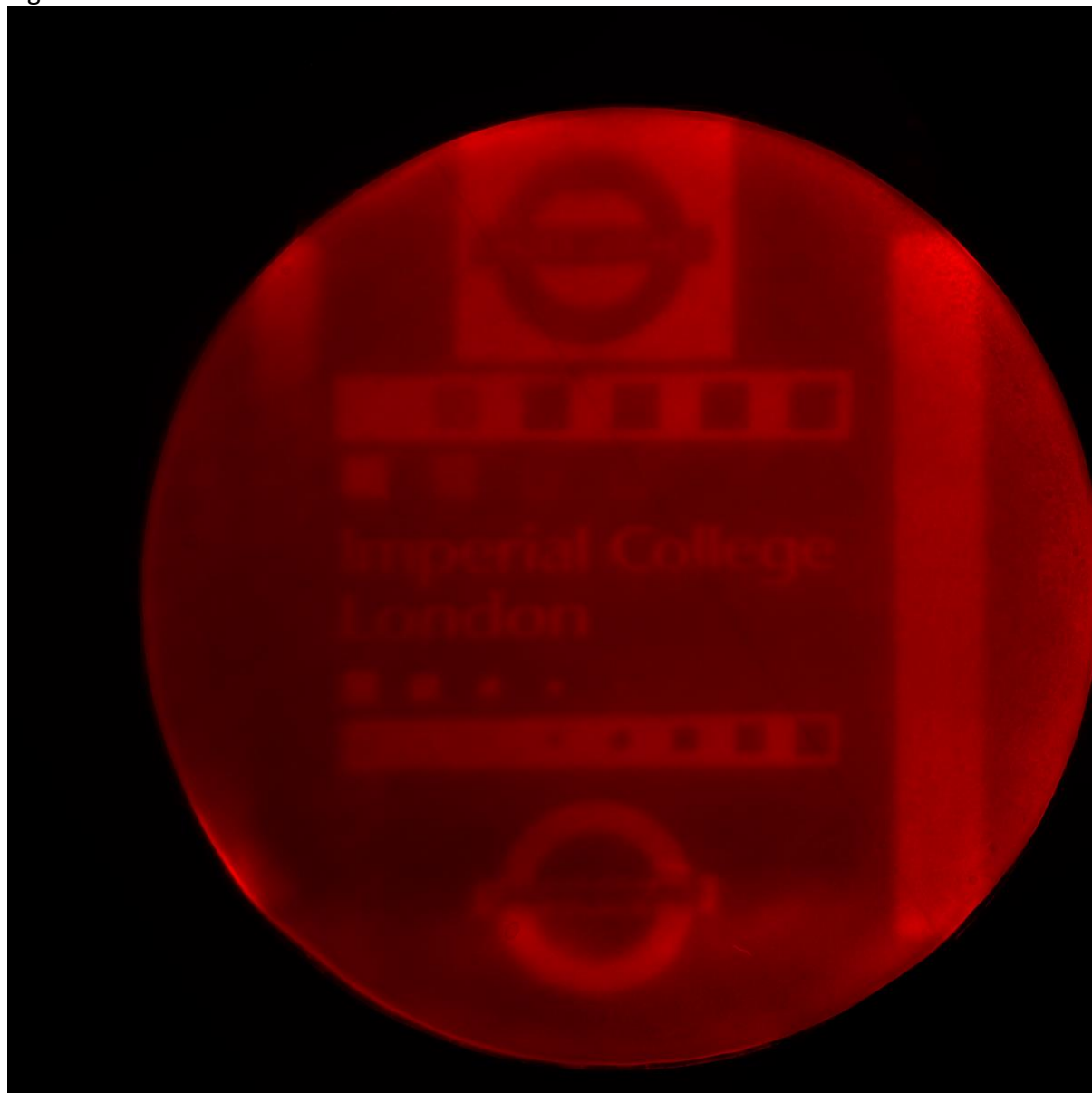

Figure 4H – Densitometry

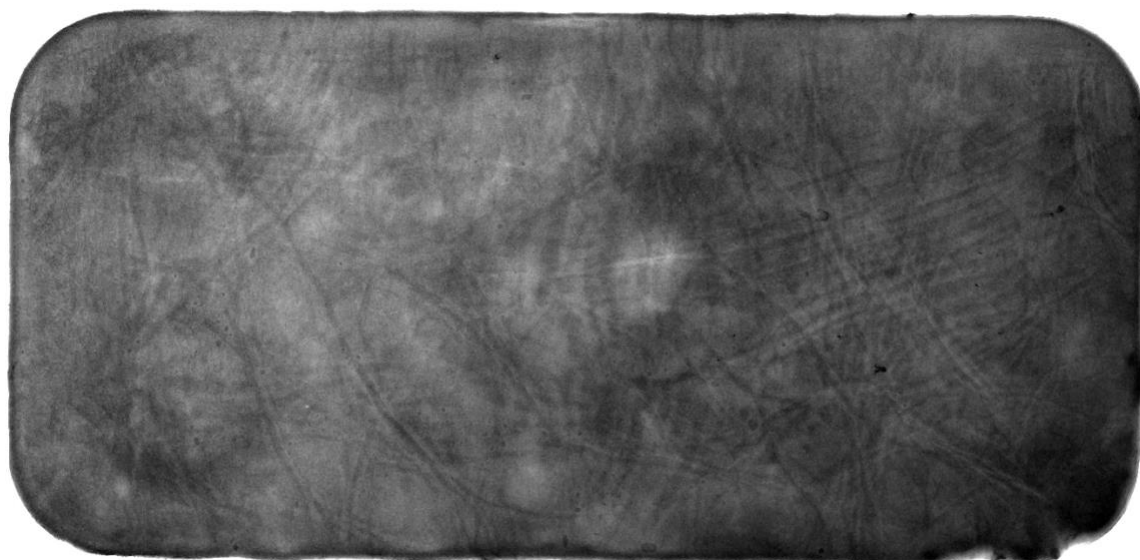

Figure 4H – Photograph

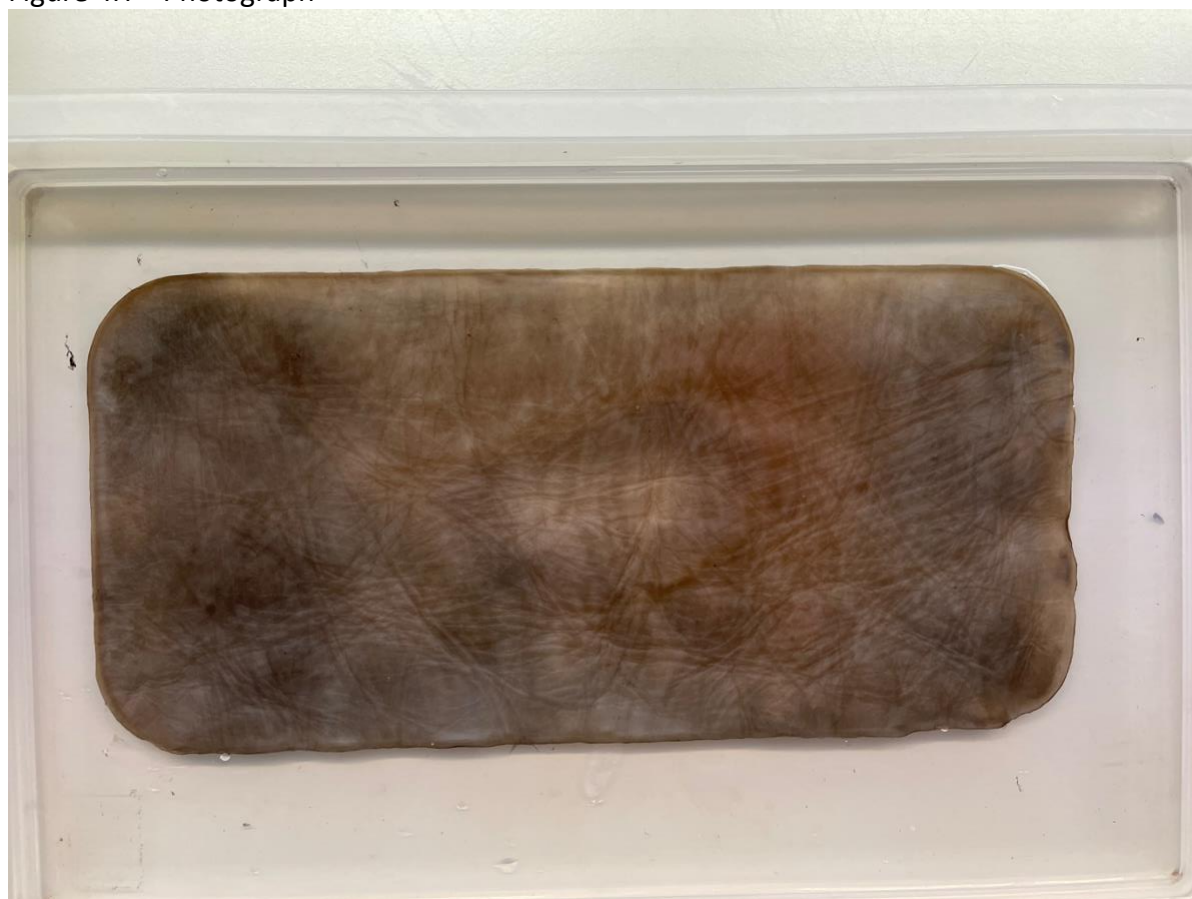

Supplement: Supplementary file 5 — Unformatted pellicle images and scans. [file 41587_2024_2194_MOESM5_ESM.pdf]
